# Methotrexate inhibition of SARS-CoV-2 entry, infection and inflammation revealed by bioinformatics approach and a hamster model

**DOI:** 10.3389/fimmu.2022.1080897

**Published:** 2022-12-21

**Authors:** Yun-Ti Chen, Yu-Hsiu Chang, Nikhil Pathak, Shey-Cherng Tzou, Yong-Chun Luo, Yen-Chao Hsu, Tian-Neng Li, Jung-Yu Lee, Yi-Cyun Chen, Yu-Wei Huang, Hsin-Ju Yang, Nung-Yu Hsu, Hui-Ping Tsai, Tein-Yao Chang, Shu-Chen Hsu, Ping-Cheng Liu, Yuan-Fan Chin, Wen-Chin Lin, Chuen-Mi Yang, Hsueh-Ling Wu, Chia-Ying Lee, Hui-Ling Hsu, Yi-Chun Liu, Jhih-Wei Chu, Lily Hui-Ching Wang, Jann-Yuan Wang, Chih-Heng Huang, Chi-Hung Lin, Po-Shiuan Hsieh, Yan-Hwa Wu Lee, Yi-Jen Hung, Jinn-Moon Yang

**Affiliations:** ^1^ Institute of Bioinformatics and Systems Biology, National Yang Ming Chiao Tung University, Hsinchu, Taiwan; ^2^ Institute of Preventive Medicine, National Defense Medical Center, Taipei, Taiwan; ^3^ Department of Microbiology and Immunology, National Defense Medical Center, Taipei, Taiwan; ^4^ Department of Biological Science and Technology, National Yang Ming Chiao Tung University, Hsinchu, Taiwan; ^5^ Institute of Molecular Medicine and Bioengineering, National Yang Ming Chiao Tung University, Hsinchu, Taiwan; ^6^ Center for Intelligent Drug Systems and Smart Bio-Devices, National Yang Ming Chiao Tung University, Hsinchu, Taiwan; ^7^ Institute of Molecular and Cellular Biology, National Tsing Hua University, Hsinchu, Taiwan; ^8^ Department of Internal Medicine, National Taiwan University Hospital, Taipei, Taiwan; ^9^ Graduate Institute of Medical Sciences, National Defense Medical Center, Taipei, Taiwan; ^10^ Institute of Microbiology and Immunology, National Yang Ming Chiao Tung University, Taipei, Taiwan; ^11^ Institute of Biophotonics, National Yang Ming Chiao Tung University, Taipei, Taiwan; ^12^ Division of Endocrine and Metabolism, Department of Internal Medicine, Tri-Service General Hospital, National Defense Medical Center, Taipei, Taiwan

**Keywords:** methotrexate, SARS-CoV-2, multi-target drugs, drug repurposing, inflammation

## Abstract

**Background:**

Drug repurposing is a fast and effective way to develop drugs for an emerging disease such as COVID-19. The main challenges of effective drug repurposing are the discoveries of the right therapeutic targets and the right drugs for combating the disease.

**Methods:**

Here, we present a systematic repurposing approach, combining Homopharma and hierarchal systems biology networks (HiSBiN), to predict 327 therapeutic targets and 21,233 drug-target interactions of 1,592 FDA drugs for COVID-19. Among these multi-target drugs, eight candidates (along with pimozide and valsartan) were tested and methotrexate was identified to affect 14 therapeutic targets suppressing SARS-CoV-2 entry, viral replication, and COVID-19 pathologies. Through the use of *in vitro* (EC_50_ = 0.4 μM) and *in vivo* models, we show that methotrexate is able to inhibit COVID-19 *via* multiple mechanisms.

**Results:**

Our *in vitro* studies illustrate that methotrexate can suppress SARS-CoV-2 entry and replication by targeting furin and DHFR of the host, respectively. Additionally, methotrexate inhibits all four SARS-CoV-2 variants of concern. In a Syrian hamster model for COVID-19, methotrexate reduced virus replication, inflammation in the infected lungs. By analysis of transcriptomic analysis of collected samples from hamster lung, we uncovered that neutrophil infiltration and the pathways of innate immune response, adaptive immune response and thrombosis are modulated in the treated animals.

**Conclusions:**

We demonstrate that this systematic repurposing approach is potentially useful to identify pharmaceutical targets, multi-target drugs and regulated pathways for a complex disease. Our findings indicate that methotrexate is established as a promising drug against SARS-CoV-2 variants and can be used to treat lung damage and inflammation in COVID-19, warranting future evaluation in clinical trials.

## Introduction

SARS-CoV-2 has spread throughout the world rapidly, infecting over 622 million people with COVID-19 and causing more than 6 million deaths, as of October 19, 2022 (https://www.who.int/). Vaccines are currently one of the most valuable tools to combat the disease, and several SARS-CoV-2 vaccines have been developed and approved to curb the spread of the virus. However, as SARS-CoV-2 mutates quickly, several variant strains have emerged that may undermine the effectiveness of the vaccines. Thus, a timely development of effective drugs for COVID-19 is an important task that could complement vaccines to protect human lives. In this regard, drug repurposing is a faster way to speed up drug discovery process as most FDA-approved drugs have well-documented profiles on the efficacy, safety, pharmacokinetics, and drug interactions (1, 2). Drug repurposing for the emerging COVID-19 pandemic has been proposed (3) and hastened with clinical studies on several approved drugs like hydroxychloroquine (4), azithromycin (5) and ivermectin (6), but their mechanisms of action are unclear and not thoroughly validated.

COVID-19 is a complex disease manifesting a myriad of symptoms and disorders ranging from mild fever and loss of taste, inflammation, to severe pneumonia, acute respiratory distress syndrome (ARDS) etc. (7) Some specific symptoms, such as thrombotic complications directly contribute to patient mortality and morbidity (8). For treating such a complex disease, multiple disorders that play a role in patient survival should be identified and simultaneously targeted to alleviate the disease and promote recovery. The primary treatment strategy for COVID-19 has been a combination of drugs such as dexamethasone and anti-interleukin-6 monoclonal antibody for anti-inflammation and remdesivir for anti-viral effects. On the other hand, multi-target drugs where one compound targets these multiple disease proteins/pathways to achieve similar therapeutic effects with less adverse effects and drug-drug interactions (due to drug cocktails) (9) appear as a very promising alternative strategy.

The drug methotrexate (MTX) acts on folate and one-carbon metabolism for *de novo* purine synthesis, and has been approved for treatment of rheumatoid arthritis (RA) (10) and cancer (11). Recent studies raised discussion on its role of COVID-19, such as whether patients receiving MTX therapy (*e.g.*, RA patients) would discontinuation on immune response after COVID-19 vaccination (12), and mechanism-based hypothesis to test MTX against COVID-19 *in vitro* (13, 14). While its mechanisms for treating COVID-19 are remaining unclear. Furthermore, whether methotrexate effectively treats COVID-19 *in vivo* and utilizing transcriptomic data remains to be established. Therefore, a systematic method for discovering multi-target drugs and mechanisms is needed to identify effective drugs for COVID-19.

In the current work, we presented an integrated drug repurposing approach with a systematic drug score (*S_drug_*) by combining structure-based Homopharma (15) and hierarchical systems biology networks (HiSBiN) (16) for screening multi-target compounds for COVID-19 from the 2,303 FDA-approved drugs. We discovered methotrexate and its multi-targeting mechanisms in treating COVID-19, which were further investigated in cells (*in vitro*) and a Syrian hamster model (*in vivo*). Additionally, methotrexate is active *in vitro* against the four major SARS-CoV-2 variants of concern (VOC). We found that methotrexate targeted and modulated multiple COVID-19 proteins/pathways, leading to suppressed SARS-CoV-2 entry, virus replication, inflammation (*via* neutrophil infiltration, neutrophil extracellular traps and regulation of immune responses), and thrombosis.

## Result

### Identifying multi-target repurposing drugs for COVID-19

**Figure 1:**
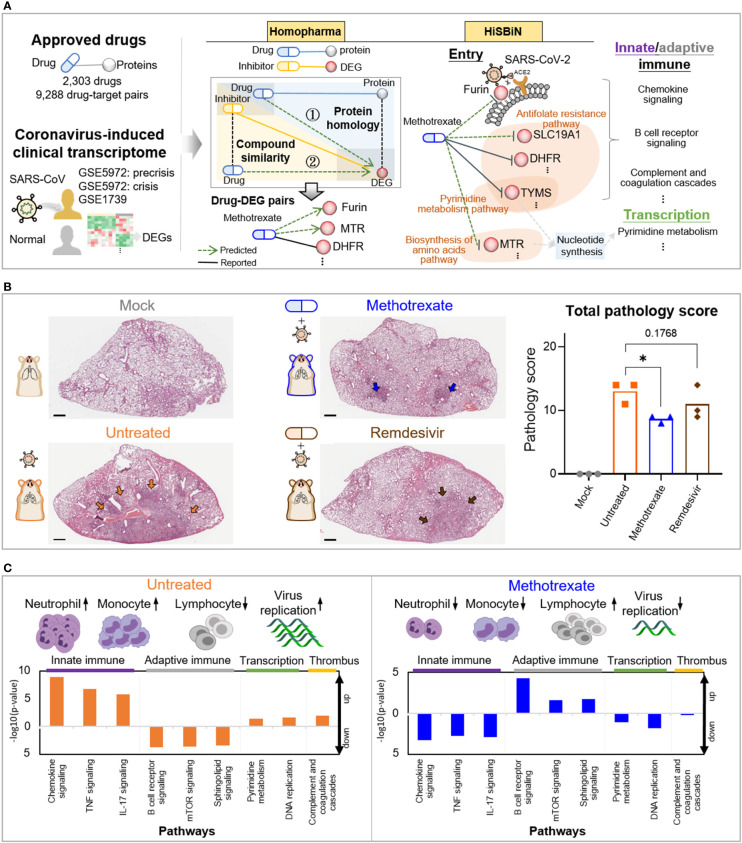
Study overview. **(A)** The steps including Homopharma, HiSBiN and the scoring system to identify multi-target repurposing drugs. The drug-protein pairs of approved drugs and the COVID-19 DEGs were obtained and input to the Homopharma which by ① protein homology and ② compound similarity, predicted drug–DEG pairs such as methotrexate with furin (of entry), MTR, DHFR, etc. The HiSBiN predicted drug perturbed COVID-19 pathways such as methotrexate interfering the pyrimidine metabolism pathway affecting the downstream pathways such as innate/adaptive immune response, transcription, etc. The green dash lines indicate the predicted drug-protein pairs (e.g., methotrexate-MTR) and their regulated pathways (e.g., Biosynthesis of amino acids pathway). **(B)** Hematoxylin and eosin (H&E)-stained lung lobes from SARS-CoV-2 Syrian hamster groups, with multifocal pulmonary inflammation (arrows) and total pathology scores. The hamster groups (n = 3) are uninfected-mock (gray), infected-untreated (orange), methotrexate-treated (blue) and remdesivir-treated (brown); scale bars are 1,000 μm, *p < 0.05. Bars indicate mean values for the four hamster groups. Statistical analysis by non-parametric Mann–Whitney test. **(C)** The systemic effects of methotrexate treatment in the infected hamsters are summarized using untreated and methotrexate-treated cases. Variations in the white blood cells (neutrophils, monocytes, and lymphocytes) and the regulation of corresponding pathways in the lung omics (e.g., innate and adaptive immune responses, transcription and thrombus) are depicted.

The workflow for multi-target anti-COVID-19 FDA drugs is summarized in **Figures 1A**, **S1**. Firstly, for the FDA drugs we inferred about 9,130 drug-protein pairs from DrugBank database. However, the reported COVID-19 drugs and targets in literature are often unobserved in the database, thus we used text-mining to collect 158 drug-protein pairs from over 140,000 literatures (**Figures 1A**, **S1A**). Also to study SARS-CoV-2 targets for therapies, we used omics analysis of two clinical coronavirus transcriptomic datasets from patients with SARS GSE5972 (precrisis and crisis) and GSE1739 (**Figures S2A, B**), given the high genome sequence identity with SARS-CoV-2, the rationale of which was validated in a recent work (17). This identified 2,516 differentially expressed genes (DEGs) of COVID-19. Next, we applied Homopharma to the input 9,130 drug-protein pairs (**Figure S1B**) to predict new drug-DEG pairs, using 162,155 inhibitor-DEG pairs from BindingDB database. This is achieved by 1) Protein homology: identifying DEG similar proteins, predict its drug and that DEG as a drug-DEG pair; 2) Compound similarity: identifying inhibitor similar drugs, predict its DEG and that drug as a drug-DEG pair, resulting in a total of 21,233 predicted drug-DEG pairs (alongside 1,335 reported drug-DEG pairs). On the other hand, HiSBiN was applied to the 2,516 DEGs to find their enriched subsystems/pathways in KEGG, to predict drug perturbation of COVID-19 pathways (with a *Z* score > 1.96). Thirdly, we formulated a systematic drug scoring to prioritize multi-target drugs, $S_{drug}=\sum_{i=1}^{T}(DT_{i})/\sqrt{T}$ , where *T* is the target number of a drug, and *DT_i_* is the score of each drug target *i*. For example, we discovered that methotrexate with a score S*_methotrexate_* = 0.52 has 14 targets and another drug pimozide with a score S*_pimozide_* = 0.8 has 56 targets. (**Figures S1C, S3C, F, I**).

**Figure 2:**
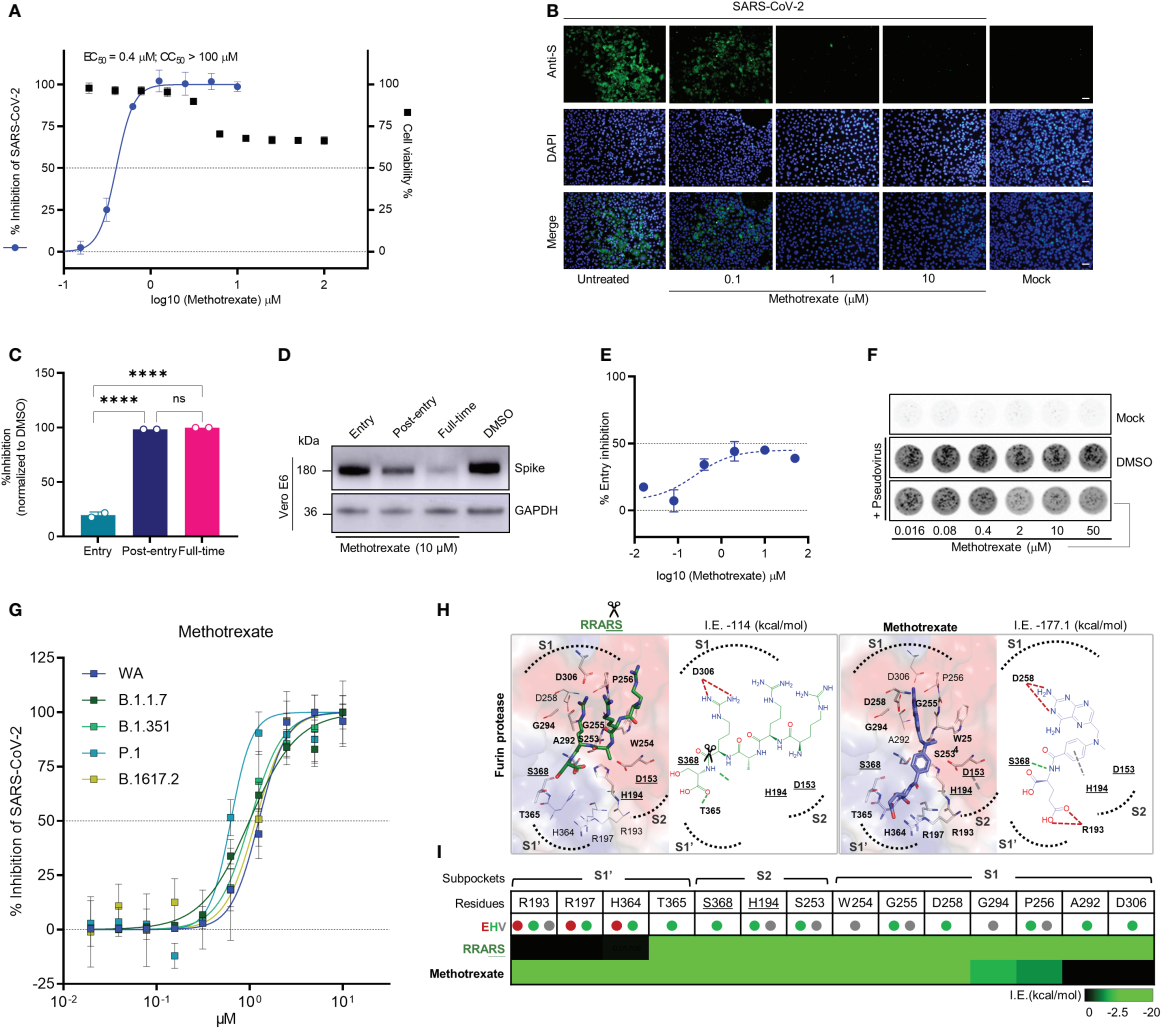
Methotrexate inhibits SARS-CoV-2 infection and entry *in vitro.* **(A)** Virus infected Vero E6 cell lines were used in: cytopathic (CPE) assay for % viral inhibition curves of methotrexate and cytotoxicity assay for % cell viability curves. The results are shown as mean ± SD of technical quadruplication. **(B)** In immunofluorescence assay, increasing doses of methotrexate caused better inhibition of SARS-CoV-2 replication, detected by anti-S Ab (green), and cell nuclei stained with DAPI (blue); scale bars are 30 μm. **(C)** Time-of-addition assay measuring the viral yield by qRT-PCR for the three time-of-addition groups. The % inhibition was calculated by the relative viral RNA expression normalized to DMSO control; statistical analyses were performed using one-way ANOVA; ****p *<* 0.0001, ns, not significant. **(D)** Western blot analysis quantifying spike protein for different time-of-addition groups, with a GAPDH control. **(E)** The virus entry inhibition by methotrexate was studied by pseudovirus neutralization assay, where the pseudovirus entering the host cell was measured by its luciferase activity. The % entry inhibition curve yields EC_50_ for entry inhibition of methotrexate. **(F)** The pseudovirus plaques for increasing concentrations of methotrexate compared to DMSO and mock. **(G)** Inhibition of wild-type (WA), B.1.1.7, B.1.351, P.1 and B.1617.2 virus variants by methotrexate in CPE assay. The horizontal dashed lines indicate 50% and 0% inhibition. Data are mean ± SD of technical octuplicate. **(H)** The binding poses with 2D diagrams of substrate spike peptide RRARS (green stick) and methotrexate (blue stick) in furin active site (surface) are shown with S1’, S1, S2 subpockets (dotted curves), catalytic triad residues (gray sticks, bold and underlined labels), key interacting residues (sticks with bold labels), and total interaction energy (I.E. in kcal/mol). In the RRARS peptide, the arg(R) interacts with the negatively charged S1 subpocket residues, the ser(S) interact with the positively charged S1’ subpocket residues, while the catalytic residues engage in arg(R)-ser(S) peptide bonds (scissors). The methotrexate pose in furin protease mimics that of the substrate peptide, with its pterin ring and carboxylic tail groups binding to negative S1 and positive S1’ subpocket residues, respectively, additionally strongly engaging S368 and H194 catalytic residues. **(I)** The interaction profile of RRARS and methotrexate in furin, with the E (electrostatic, red), H (hydrogen-bond, green) and V (van der Waals, gray) interactions.

We thus applied this scoring function to score 2,303 FDA drugs, of which the top 646 (40%) scored drugs were then clustered by their perturbation of 184 enriched COVID-19 pathways (**Figure S1D**). We obtained five clusters, from which the representative drugs for each cluster were then selected based on: a) ATC codes, b) toxicity profiles, and c) perturbation effects. Eight multi-target drug candidates were finally selected for further experimental testing. In the *in vitro* results (**Figure S4**), methotrexate showed the strongest inhibition of SARS-CoV-2 replication with a potent sub-micromolar anti-viral EC_50_ of 0.4 μM (**Figure 2A**) and thus chosen for further in *in vivo* studies.

To predict therapeutic targets, we scored the identified DEGs based on their *S_DisG_* scores determined from: (a) significant gene expression (p value) in disease vs. normal samples, (b) gene druggability (*S_druggability_*), and (c) significant gene involvement (*S_HiSBiN_*) in disease KEGG pathways and subsystems by measuring the number of interacting drugs, all of which were normalized from 0 to 1 (details in **Figure S5**). To predict COVID-19 targets, we determined the *S_DisG_* threshold (0.737) based on the precision and recall using the DisGeNET COVID-19 target set (**Figure S6A**). A final set of 327 targets for COVID-19 were predicted. Interestingly, among the top 10 COVID-19 targets scored by *S_DisG_*, eight of them, including JAK1, GSK3B, and PARP1 were recorded in the DisGeNET for COVID-19 (**Figure S6B**), and the remaining predicted targets, HDAC1 (18) and LCK (19) in independent works.

Among these predicted drug candidates, methotrexate was the most promising and could target multiple pathways including SARS-CoV-2 entry and replication as well as innate/adaptive immune response and thrombosis (**Figure 1A**). Thus methotrexate was further evaluated using *in vitro* and *in vivo* COVID-19 models (20, 21). In the animal studies, we compared virus infection, white blood cells (WBCs), histopathology, pathology scoring and immunohistochemistry (IHC) among hamster groups (uninfected mock, infected-untreated, methotrexate-treated, and remdesivir-treated). Representative images of hematoxylin and eosin (H&E)-stained lungs and total pathology scores are shown in **Figure 1B**. Furthermore, WBC analysis and omics data analysis of RNA sequencing obtained from lung tissues revealed detailed systemic effects and multi-targeting mechanisms of methotrexate and underlying genes and pathways involved (**Figure 1C**). Methotrexate suppressed overall viral replication, decreased neutrophils and monocytes in agreement with regulation of innate immune pathways, increased levels of lymphocytes related to adaptive immune pathways, and modulation of transcription and thrombus pathways. These results show that our repurposing approach can identify therapeutic targets, multi-target drugs and regulated pathways for an emerging complex disease.

### Methotrexate inhibited the replication and entry of SARS-CoV-2 and variants of concern

The drug candidates were experimentally tested by *in vitro* antiviral cytopathic effect (CPE) and cytotoxicity assays, along with the reference drug remdesivir. In the CPE assay, Vero E6 cells were inoculated with SARS-CoV-2 (multiplicity of infection [MOI] of 0.001) and treated with solvent or various doses of respective drugs and incubation for 48 h to measure the dose-dependent % virus inhibition. Cytotoxicity studies were also conducted to evaluate the % cell viability for solvent or various drug dose treatments. From the *in vitro* results (**Figure S4**), methotrexate showed the strongest inhibition of SARS-CoV-2 replication with a potent sub-micromolar anti-viral EC_50_ of 0.4 μM (**Figure 2A**) which was significantly better than that of the remdesivir (EC_50_ of 12.47 μM) (**Figure S4H**). Additionally, methotrexate was relatively non-cytotoxic with a CC_50_ > 100 μM and thus attained a selectivity index (SI = CC_50_/EC_50_) of ~250. Methotrexate’s dose-dependent inhibition of virus replication was further confirmed using an immunofluorescence assay where the SARS-CoV-2 replication in infected host cells was visualized by a fluorescent-tagged antibody against the spike protein of the virus. As shown in **Figure 2B**, methotrexate decreased green florescence and thus the virus count in a dose-dependent fashion; only very rare green fluorescence was noted in cells treated with 1 μM of methotrexate and become completely absent in cells treated with 10 μM of methotrexate. These results indicate methotrexate is a non-cytotoxic yet potent drug against SARS-CoV-2.

To investigate the mechanisms of SARS-CoV-2 inhibition by methotrexate, we performed a time-of-addition assay in which Vero E6 cells inoculated with SARS-CoV-2 (MOI of 0.01) were treated with methotrexate at various addition times as following: ‘entry’ - drug added simultaneously with the virus and removed 1 h post-infection, ‘post-entry’ - drug added at 1 h post-infection and incubated for 24 h, ‘full-time’ - drug added with the virus and incubated for 24 h. The amount of virus was quantified and % virus inhibition was calculated for each group. The strongest inhibition of virus replication was observed when methotrexate was added post-entry and for full-time (nearly 100%), while moderate inhibition (~20%) was noted when methotrexate was added at the entry stage (**Figure 2C**). We validated the results by western blot, where the virus spike protein was detected in infected Vero E6 cells in different time-of-addition groups (**Figure 2D**). A slight decrease in spike protein expression in the entry group compared to DMSO control was observed, followed by further reduction in post-entry and full-time groups. The inhibition of viral entry by different doses of methotrexate was further explored by an *in vitro* pseudovirus neutralization assay. Entry of SARS-CoV-2 spike-based pseudovirus was measured by the intracellular luciferase activity encoded in the genome. In addition, virus plaques formed were also quantified. We found methotrexate dose-dependently decreased viral plaques and determined the EC_50_ of entry inhibition as 76.30 μM (**Figures 2E, F**). It must be noted that, furin inhibition could lead to a maximal inhibition of ~50% overall virus entry, as residual viral entry is still possible by other mechanisms.

The SARS-CoV-2 virus mutates quickly and four major VOCs namely B.1.1.7 (Alpha), B.1.351 (Beta), P.1 (Gamma), and B.1.617.2 (Delta) have emerged since the outbreak of the pandemic (22). Thus, it is important to develop therapies and drugs along with vaccines, to combat these virus variants. As our results indicate methotrexate potently inhibited SARS-CoV-2 (WA), we were interested to further investigate whether methotrexate effectively inhibits other variants. We employed the CPE assay where Vero E6 cells were inoculated with the four SARS-CoV-2 variants (MOI of 0.001) followed by treatments with various doses of methotrexate and incubation for 72 h. The % virus inhibition were then plotted against the drug concentrations for all four variants (**Figure 2G**). The results showed methotrexate potently inhibited the replication of all four variants in dose-dependent manner with EC_50_ values of 1 μM for B.1.1.7, 1 μM for B.1.351, 0.63 μM for P.1, and 1.16 μM for B.1.617.2, which are in similar range as for WA (1.25 μM). These results strongly support methotrexate could treat all the four VOCs and would be a highly valuable drug against mutating SARS-CoV-2.

Several previous studies have established that the furin protease, alongside TMPRSS2, is a key human host protease essential for the viral spike protein maturation and entry into host cells (23), thus making furin an important anti-COVID-19 drug target (24, 25). According to our predictions and scores, methotrexate could target furin, based on its high compound similarity (*C.S. =* 0.806) with folic acid which was reported to target furin (24). Thus we explored mechanisms of furin inhibition by methotrexate, specifically to block furin-mediated cleavage of specific substrate peptide motif RRARS (26) at SARS-CoV-2 spike S1/S2 site, necessary for RBD opening and binding to the host cell ACE2 for viral entry (**Figure S3A**). The targeting of furin protease by methotrexate was confirmed using an enzymatic assay (24), where 100 μM of methotrexate competitively inhibited ~73% of furin protease activity, while similar compound folic acid showed< 30% inhibition. We additionally used a structure-based analysis to elucidate the mechanisms of furin inhibition by methotrexate, folic acid and the RRARS substrate peptide. We observed that the substrate peptide RRARS occupied key furin S1, S1’ and S2 charged subpockets (dotted curves, surface positive: blue, negative: red) where substrate arg(R) interacted with D306 at S1, and substrate ser(S) engaged T365 at S1’ of furin (**Figures 2H, I**). Methotrexate displayed a similar binding pose in furin, being strongly bound to these subpockets with its charged positive ring group engaging D258 at S1 and negative tail group interacting with R193 at S1’. In addition, methotrexate strongly interacted with the catalytic residues (underlined labels), S368 by hydrogen-bond and H194 by van der Waals interactions. We further examined how the methotrexate’s binding in a new target, by analysis of methotrexate and folic acid binding in DHFR vs. furin (**Figure S3A**). These combined *in vitro* and *in silico* results support methotrexate’s inhibition of SARS-CoV-2 entry by targeting furin.

Inhibition of DHFR by methotrexate leads to blockade of folate synthesis (and nucleic acid) (27). Adding folinic acid to methotrexate treated cells rescues the cells by replenishing folate reserves, a process referred to as folinic acid rescue (28). Here, we used folinic acid rescue to study the role of DHFR inhibition by methotrexate in the suppression of virus replication (**Figure S7**). We found methotrexate significantly blocked virus replication and this inhibition was reversed and rescued by addition of folinic acid (**Figure S7A**). These data suggest DHFR inhibition and depletion of folic acid is an underlying mechanism by which methotrexate inhibited virus replication. Western blot analysis (**Figure S7B**) indicated the expression of spike protein could be rescued by folinic acid in cells treated with methotrexate, further validating DHFR inhibition (and depleted folate and nucleic acid synthesis) as a key mechanism of methotrexate to inhibit SARS-CoV-2 replication.

### Evaluating methotrexate in SARS-CoV-2-infected Syrian hamsters

**Figure 3:**
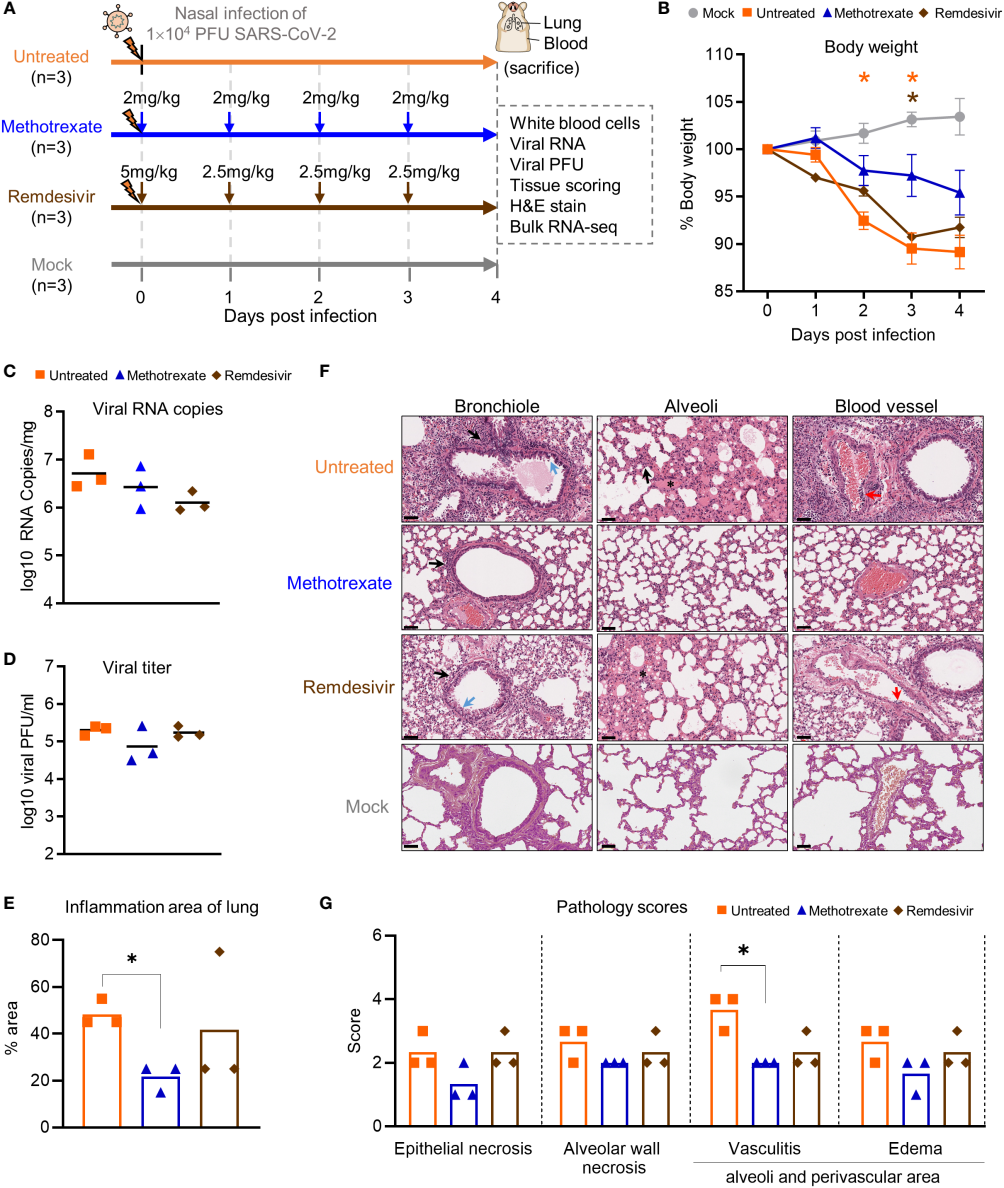
Methotrexate inhibits lung infection, damage and inflammation in SARS-CoV-2-infected hamsters. **(A)** Study design: Twelve hamsters were divided into 4 hamster groups (each group, n=3), of which 3 groups were infected with SARS-CoV-2 (10^4^ PFU) intranasally. For the 2 treatment groups, from the day of infection a daily administration of methotrexate (2 mg/kg) and remdesivir (5 mg/kg at day 0 then 2.5 mg/kg) were given for 4 dpi. On 4 dpi, all the hamsters were sacrificed and the samples collected. **(B)** Body weight analysis for methotrexate-treated (blue), remdesivir-treated (brown), untreated (orange) and mock (gray) hamster groups. Statistical analyses were performed using two-way ANOVA, Tukey’s post-test, with the p value compared to the mock. The results are shown as mean ± SD. **(C)** The virus load in hamster lungs was by quantified by qRT-PCR of virus RNA copies. Statistical analyses were performed using non-parametric Kruskal–Wallis test; p value of untreated vs. methotrexate ≥ 0.9999 and untreated vs. remdesivir = 0.3255. **(D)** Viral titers in the lung samples measured by the plaque-forming units (PFU/ml). Statistical analyses were performed using the non-parametric Kruskal–Wallis test; p value of untreated vs. methotrexate = 0.8206 and untreated vs. remdesivir ≥ 0.9999. **(E)** Quantification of the total inflammation area of the lung of the untreated, methotrexate-treated and remdesivir-treated groups. **(F)** Representative H&E-stained magnified images of bronchioles, alveoli and blood vessels for the four hamster groups; scale bars are 50 μm. In the bronchioles, bronchial epithelial cell degeneration and necrosis (blue arrow) and peribronchiolar mononuclear cell infiltration (black arrow) are shown. In the alveoli, alveolar necrosis with massive alveolar space infiltration (black arrow) and pulmonary edema (asterisk) are shown, while in the blood vessels the vasculitis (red arrows) is highlighted. **(G)** Pathology scores for individual parameters. Statistical analyses of **(E, G)** were performed using the non-parametric Mann–Whitney test, *p < 0.05.

After determining the antiviral activities of methotrexate *in vitro*, we evaluated the efficacy of methotrexate *in vivo* using Syrian hamsters, which is a well-established COVID-19 animal model for studying the pathogenesis and treatments for COVID-19 (20). Methotrexate is a well-studied drug for rheumatoid arthritis, with suitable pharmacokinetics showing a relatively rapid absorption (29). In the present study, hamsters were divided into uninfected mock, infected untreated control, infected methotrexate-treated, and infected remdesivir-treated groups (n = 3). For virus infection hamsters were intranasally inoculated 10^4^ plaque-forming units (PFU) of SARS-CoV-2. The infected hamsters developed clinical signs of lethargy, ruffled fur, and anorexia starting from the 1^st^ day post infection (dpi). For the drug treatment groups, a loading dose of 2 mg/kg methotrexate or 5 mg/kg remdesivir was given on the day of infection, then a daily dose of 2 mg/kg methotrexate or 2.5 mg/kg remdesivir was given on 1^st^ to 3^rd^ dpi (**Figure 3A**). The hamsters were sacrificed on 4^th^ dpi, the blood samples were taken for cell analyses, and the lungs were collected for viral RNA and PFU quantification, RNA sequencing, histopathology and IHC studies.

In the initial body weight analysis, we observed that the weight loss was alleviated in all three methotrexate-treated hamsters, compared to the untreated hamsters (**Figure 3B**). This effect was better than that of remdesivir treatments, indicating a better health status attained by methotrexate treatments. Next, the viral load in the lungs of hamsters was quantified by qRT-PCR. We found methotrexate decreased virus RNA copies as compared to the untreated group (**Figure 3C**). Furthermore, a plaque assay was used to measure the infectious virus in lungs (**Figure 3D**), where methotrexate treatment reduced plaque counts by about 10-fold in two hamsters whereas remdesivir seemed ineffective.

**Figure 4:**
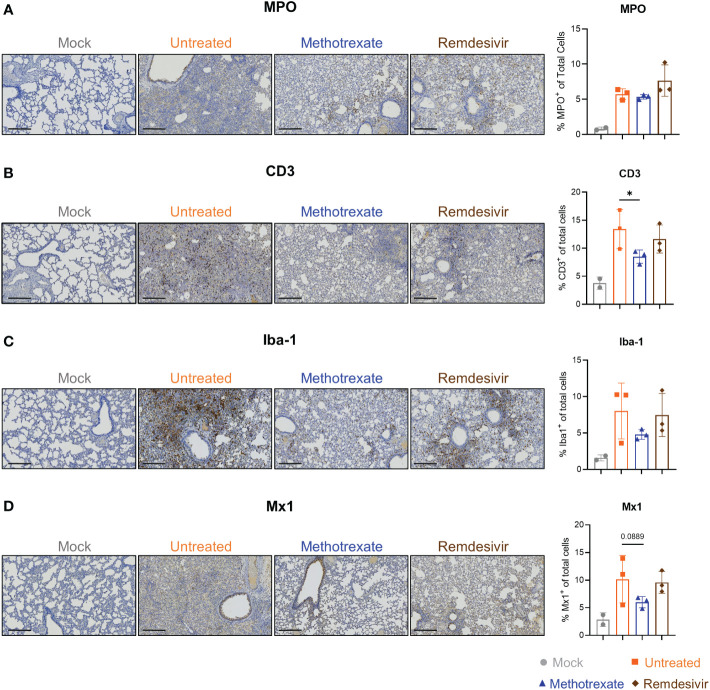
Immunohistochemistry (IHC) studies showed that methotrexate affected the innate/adaptive immune responses in hamster lungs. Methotrexate reduced neutrophil and macrophage infiltration in hamster lungs. The representative immunohistochemistry (IHC) images and quantifications of **(A)** neutrophils (myeloperoxidase^+^, MPO, % MPO^+^ of total cells), **(B)** macrophages (ionized calcium-binding adaptor molecule 1^+^, Iba-1, % Iba-1^+^ of total cells) **(C)** T cell surface glycoprotein^+^ (CD3, % CD3^+^ of total cells), and **(D)** interferon-induced GTP-binding protein^+^ (Mx1, % Mx1^+^ of total cells), in lungs of methotrexate-treated (blue), remdesivir-treated (brown), untreated (orange) and mock (gray) hamster groups. Scale bars in IHC images are 300 μm. Statistical analysis was performed by an unpaired Student’s *t*-test where drugs-treated groups were compared with untreated group, *p *<* 0.05.

To further assess the effectiveness of methotrexate in ameliorating SARS-CoV-2 infection and pathologies in the hamster lungs, histopathological examination on the H&E-stained lung sections (**Figure 1B**) were performed independently by two pathologists in a blinded fashion. Methotrexate decreased an overall ~30% inflammation area in the lungs in all three hamsters compared to the untreated, an efficacy that was better than remdesivir (**Figure 3E**). Detailed pathologies in bronchioles, alveoli, blood vessels are shown in **Figure 3F**, followed by a summary of the pathology scores in **Figure 3G**. In the untreated hamsters, marked bronchial epithelial cell necrosis (blue arrows) with peribronchiolar inflammatory mononuclear cell infiltration (black arrow) was observed; alveolar wall necrosis, edema (asterisk), alveolar space infiltration (black arrow), and vasculitis (red arrows) were also evident. On the contrary, lung histology of the methotrexate-treated hamsters were largely similar to those of the uninfected mock group (**Figure 3F**). Little infiltration by inflammatory cells in the bronchiole and alveoli along with mild vasculitis was noted, suggesting recovery to a healthier state after methotrexate treatment. In the remdesivir group, improved inflammation, a mild degree of bronchiolar epithelium cell death, peribronchiolar mononuclear cell infiltration, and a severe degree of pulmonary edema, were observed. Detail histopathological examinations indicate methotrexate improved epithelial necrosis (with or without inflammatory cell infiltration of the bronchiole), alveolar wall necrosis and vasculitis and edema (in alveoli and perivascular area), often better than remdesivir in alleviating these pathologies. Consistent with the histological examination with H&E-stained sections, quantitative IHC studies of hamster lung tissues (**Figure 4**) showed levels of activated neutrophils (MPO^+^), macrophages (Iba-1^+^), T cells expressing the inflammation markers (CD3^+^ and Mx1^+^), were decreased in the lungs of methotrexate-treated hamsters. In summary, histopathology and IHC studies indicate methotrexate is an effective drug for reducing SARS-CoV-2 infection, inflammation and pathologies.

### Hematological analysis of methotrexate-treated SARS-CoV-2-infected hamsters

**Figure 5:**
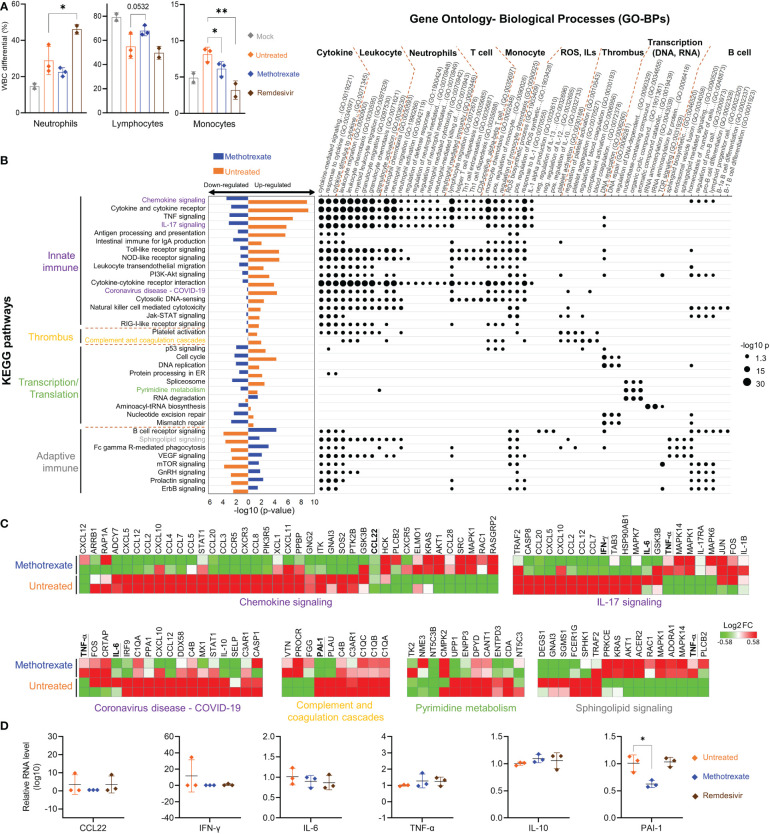
Methotrexate modulated gene expression, pathways and biological processes of inflammation, immune responses, transcription and thrombosis in SARS-CoV-2 infected hamsters. **(A)** Analysis of populations of white blood cells (WBC) such as neutrophils, lymphocytes and monocytes for mock (gray), infected-untreated (orange), methotrexate-treated (blue) and remdesivir-treated (brown) hamster groups. Bars indicate mean values. Statistical analysis was performed by an unpaired Student’s *t*-test, drug-treated groups compared with untreated group, *p*<* 0.05, **p*<* 0.01. **(B)** Enriched KEGG pathways for untreated (control vs. infected untreated hamsters) and methotrexate-treated cases (infected vs. methotrexate-treated hamsters). For each pathway, bar graphs show significant upregulated and downregulated genes, that were reversed between untreated (orange bars) and methotrexate-treated cases (blue bars). The pathways are categorized into innate immunity, adaptive immunity, thrombosis and transcription/translation. The enriched gene ontology-biological processes (GO-BPs) for each pathway gene set are shown as a dot plot matrix, with black dot sizes representing the significance by –log (p value), and similar biological processes clustered. See **Table S1** for full names of KEGG pathways and GO-BPs. **(C)** For specific pathways from each category, the gene expression fold change are shown as heat maps, for untreated and methotrexate-treated cases (red: upregulated; green: downregulated), some genes of interest bold and underlined. **(D)** For specific genes, qRT-PCR-based validation of gene expressions in lung samples from three hamster groups. The results are shown as mean ± SD. Statistical analyses were performed by the non-parametric Mann–Whitney test, *p < 0.05.

We explored the mechanisms and processes underlying methotrexate’s anti-viral and anti-inflammatory activities in SARS-CoV-2-infected hamsters. We first analyzed various WBC populations in the blood of mock, untreated, methotrexate-treated and remdesivir-treated hamsters, the results showing levels of neutrophils, lymphocytes, and monocytes are in **Figure 5A**. We observed significantly decreased neutrophils and monocytes in blood upon methotrexate treatments compared to the untreated group, and this efficacy was better than that of remdesivir. Lymphopenia is an important phenomenon observed during severe SARS-COV-2 infection (30). In this regard, methotrexate treatment significantly increased blood lymphocyte count compared to that of the untreated group, which may be beneficial in countering COVID-19 lymphopenia.

### Omics analyses and bioassay evaluations in the hamster model reveal multi-targeting by methotrexate

Parts of the hamster lungs were subjected to RNA sequencing using Illumina platform, followed by whole-genome transcriptome analysis. The sequence data are deposited in the National Center for Biotechnology Information Gene Expression Omnibus under accession code GSE179709. In RNA-seq analysis, a fold change of ≥ 2.0 was set to identify 710 upregulated and 582 downregulated DEGs in infected vs. control samples, and 489 upregulated and 621 downregulated DEGs in methotrexate-treated vs. infected samples, followed by KEGG pathway analysis and functional analysis for affected ontology biological processes (GO-BPs) (**Figure 5B**). The systemic effects of methotrexate treatment and underlying mechanisms were explored. The enriched KEGG pathways in methotrexate-treated and untreated cases, and their significant down/upregulation are shown, along with enriched gene GO-BPs of DEGs from each pathway.

In SARS-CoV-2-infected hamsters, methotrexate’s perturbations of the enriched KEGG pathways and GO-BP terms, along with the respective expressions of key genes further validated by IHC (**Figure 4**) studies and qRT-PCR (**Figure 5**) are summarized as follows: a) The enriched KEGG pathways, of which the gene expression in the diseased state was reversed by methotrexate, are primarily grouped into four categories: innate immune responses (purplelabel, IL-17 signaling, IL-6 and IFN-γ), transcription (green label, pyrimidine metabolism), adaptive immune responses (gray label, B cell receptor signaling, CD3^+^ T cells in **Figure 4B**) and thrombosis (yellow label, complement and coagulation cascades, PAI-1). b) The enriched GO-BP functional processes can be roughly clustered into several types, related to cytokines, thrombus and transcription, and neutrophils (MPO^+^ in **Figure 4A**), T/B cells (lymphocytes) and monocytes which were observed to be perturbed in the blood cell analysis results (**Figure 5A**). c) Most of the enriched KEGG pathways and their genes are in consensus with biological processes of GO-BP terms, such as chemokine/cytokine receptor/IL-17 signaling pathways with IL-6/IFN-γ/IL-10 genes are highly related to cytokine and leukocyte processes (**Figure 5B**); DNA replication/pyrimidine metabolism pathways are in direct connection to transcription; complement and coagulation cascades pathway with PAI-1 gene are linked to GO-BP thrombus process. These results imply that methotrexate has multi-targeting properties to modulate innate and adaptive immune responses, virus transcription/replication, and thrombosis in SARS-CoV-2 infected Syrian hamsters.

### Methotrexate modulated innate and adaptive immune responses in SARS-CoV-2-infected hamsters

Among the various enriched pathways affected by methotrexate, the innate immune response-related pathways and genes were significantly upregulated in the untreated case. Methotrexate treatment reversed the pattern (bar charts on the left side, **Figure 5B**) to downregulate genes in most of these pathways associated with SARS-CoV-2 infection (31), like innate immune and ROS generation (32). For instance, methotrexate treatment significantly modulated pathways of chemokine signaling, cytokine-cytokine receptor interaction, TNF signaling, IL-17 signaling and Toll-like receptor signaling pathways associated with COVID-19 immunology (33). Here, we observed downregulation of major cytokines and chemokines such as IFN-γ and IL-6 in these pathways (underlined genes in **Figure 5C**). These cytokines and chemokines were further validated by qRT-PCR (**Figure 5D**), indicating that suppression of the immune response pathways by methotrexate could mitigate COVID-19 symptoms, related to hyper-inflammation and cytokine storm.

The second major type of pathways modulated by methotrexate treatment were related to the adaptive immune response, such as B cell receptor signaling, sphingolipid signaling pathway and mTOR signaling pathways, resulting in activation of B cells and T cells to counter viral infection (**Figure 5B**). The B cell receptor signaling pathway upregulated by methotrexate involves genes with enriched biological processes related to B cell differentiation. Sphingolipid signaling is another key adaptive immune pathway up-regulated by methotrexate leading to increased sphingosine-1-phosphate (S1P) which acts as a lipid signaling molecule that binds with S1P receptors to regulate B cell circulation (34). Methotrexate also significantly upregulated other adaptive immune pathways such as mTOR signaling which is a central regulator of T cell responses in fighting infections (35). These observations on the modulation of adaptive immune pathways by methotrexate treatment are in agreement with the IHC studies in **Figure 4**. Here in the methotrexate-treated hamster group, activated T cells were decreased in lung tissues (% CD3^+^ T cells) compared to the untreated group (**Figure 4B**), which is in line with decreased T cell diapedesis and extravasation GO-BPs modulated by methotrexate. Additionally, an overall decreased virus replication upon methotrexate treatment in hamster lungs may lead to lesser adaptive immune responses.

### Methotrexate treatment suppressed neutrophil infiltration and inflammatory cytokines in SARS-CoV-2-infected hamsters

Most of the methotrexate-downregulated innate immune pathways observed in our analysis such as the chemokine, cytokine receptor, TNF and IL-17 signaling pathways have been reported to be involved in hyper-inflammation, a hallmark of COVID-19 disease (31). Upon exploring the enriched biological processes (**Figure 5B**) related to these methotrexate-modulated pathways, we identified neutrophil activation, chemotaxis & migration, Th1 lymphocyte migration & diapedesis and monocyte chemotaxis, which were in strong agreement with the overall decrease in neutrophils in the blood upon methotrexate treatment (**Figure 5A**). Also in the histopathology and IHC examination results methotrexate decreased MPO^+^ neutrophils (**Figure 4A**) due to reduced neutrophil infiltration, decreased Iba-1^+^ macrophages (related to monocytes) due to reduction in their infiltration (**Figure 4C**), which strongly validated the omics-based inferences. These results conclusively indicate that methotrexate led to reduction of neutrophils and monocytes in the lungs to achieve its anti-inflammation activity. Additionally, methotrexate modulated adaptive immune response pathways such as the B cell receptor signaling pathway involving genes with enriched GO-BP of B cell differentiation, which were in line with increased lymphocyte levels and migration (improving lymphopenia) and enhanced extravasation. The sphingolipid pathway was another adaptive immune pathway significantly modulated by methotrexate treatment with upregulated genes in treated case compared to untreated (**Figure 5C**). As this pathway played a role in COVID-19 and its stimulation hypothesized as a potential strategy in combating SARS-CoV-2 associated hyper-inflammation (36), methotrexate may reduce inflammation by enhancing sphingolipid pathway.

We further explored the anti-inflammatory effects of methotrexate by examining its modulation on cytokines and chemokines. For instance, the enriched IL-17 signaling pathway is responsible for the production of IL-17, which recruits neutrophils, monocytes, and macrophages and induces other cytokines, such as IL-6 and IFN-γ. Downregulation of this pathway by methotrexate (**Figure 5B**) led to the suppression of neutrophil and monocyte migration and significant downregulation of IFN-γ and IL-6 which were further validated by qRT-PCR (**Figure 5D**) providing effects comparable to those of remdesivir. Another pathway modulated by methotrexate is chemokine signaling, which plays a key role in COVID-19 patients exhibiting strong chemokine-dominant hypercytokinemia, where expression of pro-inflammatory cytokines, chemokines and typical antiviral ISG genes are upregulated (31). Here, methotrexate downregulated expressions of chemokines CCL2, CCR5, CCL7, CCL12, CXCL10 (**Figure 5C**) and CCL22 suppression confirmed by qRT-PCR (**Figure 5D**) leading to an anti-inflammatory effect. Furthermore, direct evidence from qRT-PCR showed methotrexate treatment increased anti-inflammatory cytokine IL-10 compared to untreated, directly contributing to its anti-inflammatory activity. Thus, methotrexate modulates multiple COVID-19-associated chemokines, cytokines and pathways for its strong anti-COVID-19 inflammation effects.

The overall effects of methotrexate in COVID-19 are positive by affecting multiple cell types, inflammatory pathways and cytokines/chemokines; however, the underlying targeting mechanisms should be connected to its effects. For instance, methotrexate has been reported to suppress inflammation *via* increased adenosine release to inhibit neutrophil extracellular trap (NET) formation in RA (27), thus methotrexate can be expected to reduce NETs formation in COVID-19 (5) for its anti-inflammatory effects. On the other hand, lymphopenia observed in the infected hamsters strongly correlated with the increased levels of IL-1β, IFN-γ, CXCL10, and CCL2, indicating a dysregulation of T-helper-1 (Th1) cell function (37). Methotrexate downregulated IFN-γ, and modulated Th1 related biological processes to restore lymphocyte count (**Figure 5A**). Methotrexate improved lymphopenia, a marker of COVID-19 disease severity (30), and thus increased the overall ability to eradicate the virus in infected lungs (**Figure 3F**). These observations are supported by previous studies where methotrexate modulated the balance of Th1 pro-inflammatory and Th2 anti-inflammatory cytokines (27, 38). In summary, the suppression of neutrophils and monocytes and their activation/migration and improvement of lymphopenia in SARS-COV-2 infected hamsters may account for the mechanism of methotrexate in treating COVID-19 infection and pathogenesis.

We also explored the connections between key histopathological features (**Figure 3**) and omics results (**Figure 5**) for the anti-COVID-19 mechanisms of methotrexate. The infection of alveolar epithelial cells by SARS-CoV-2 in hamsters results in the development of ARDS (also observed in human patients) (39), which is due to the death of alveolar epithelial cells (untreated group in **Figure 3F**) and release of pro-inflammatory mediators and cytokines. Pathway/gene/biological process analyses aid in the understanding of tissue-level pathologies, such as the alveolar epithelial cell necrosis observed in infected hamster lung tissues (**Figure 3G**) and its amelioration by methotrexate. Here, methotrexate regulated the expression of enriched COVID-19 KEGG pathway in untreated case (**Figure 5B**). From the enriched COVID-19 pathway, we could postulate that that alveolar cell necrosis released damage-associated molecular patterns (DAMPs) which in turn triggered the innate immune response *via* the Toll-like receptor pathway (40) and eventually led to cytokine storm and hyper-inflammation. Methotrexate decreased alveolar cell necrosis with a better potency than remdesivir (**Figure 3G**), which would likely reduce DAMPs and decrease triggering of the TLR pathway. This notion is supported by the finding that, the upregulated TLR pathway in the untreated hamsters was reversed in the methotrexate-treated hamsters. In summary, omics analysis and histopathological results show that methotrexate treatment is able to modulate chemokine, cytokine receptor and IL-17 signaling pathways to suppress neutrophil infiltration and inflammatory cytokines in SARS-CoV-2-infected hamsters.

### Methotrexate reduced virus replication in SARS-CoV-2-infected hamsters

Methotrexate treatment modulated pathways pertaining to nucleic acid synthesis and transcription/translation, including the cell cycle, DNA replication, pyrimidine metabolism, and nucleotide repair pathways enriched to reduce SARS-CoV-2 replication (**Figures 3C, D**). The suppression of pyrimidine metabolism by methotrexate observed here (**Figure 5C**), was well-established by its direct inhibition of DHFR involved in folate biosynthesis (**Figure S7**) and TYMS involved in pyrimidine biosynthesis (27), which are critical processes for essential building blocks in virus RNA replication. Additionally, methotrexate also downregulated protein processing in the endoplasmic reticulum (ER) pathway which could affect virus protein production. This in turn counteracts hijacking of the ER by SARS-CoV-2. Methotrexate also downregulated the spliceosome pathway (**Figure 5B**) and the accumulation of spliceosomes, which is induced by inhibition of global mRNA splicing by viral NSP16 during SARS-CoV-2 infection (41). In summary, methotrexate affected biological processes related to the central dogma of the cell to reduce SARS-CoV-2 replication.

### Methotrexate affected thrombus-related processes in SARS-CoV-2-infected hamsters

Furthermore, methotrexate affected the complement and coagulation cascades and platelet activation pathways (**Figure 5B**), which are involved in thrombus-related processes and events such as platelet aggregation and blood coagulation. The formation of thrombi and their pathological role in COVID-19 inflammation, and organ failure have been well-established (42, 43). In this regard, elevated levels of Plasminogen Activator Inhibitor-1 (PAI-1) have been reported to play a key role in COVID-19-associated coagulopathy, and its targeting have therapeutic benefits in COVID-19 (44). Here, we observed that methotrexate significantly repressed these upregulated pathways and suppressed the gene expression of PAI-1 (**Figure 5C**). qRT-PCR validation (**Figure 5D**) further confirmed that PAI-1 was significantly suppressed by methotrexate in the lungs of infected hamsters, while the standard drug remdesivir showed no effect against PAI-1 expression. This unique therapeutic effect of diminishing thrombus formation is an unexpected advantage, as no other treatments have been reported to achieve this protection. Therefore, methotrexate might be an important drug to improve morbidity and mortality in severely infected COVID-19 patients.

In summary, we integrated omics analysis, cell population analysis and qRT-PCR to comprehensively elucidate and validate the cellular, histopathological and system-level processes occurred in SARS-CoV-2 infected hamsters and affected by methotrexate treatment. These results conclusively support the multi-targeting action of methotrexate including overall regulation of innate/adaptive immune responses, transcription/translation, and thrombus pathways to mediate its anti-viral and anti-inflammation effects in combating SARSCoV-2 infection. Our findings suggest that methotrexate is a promising drug against SARS-CoV-2 and its VOCs, warranting further clinical trials in human patients in the future.

## Discussion

**Figure 6:**
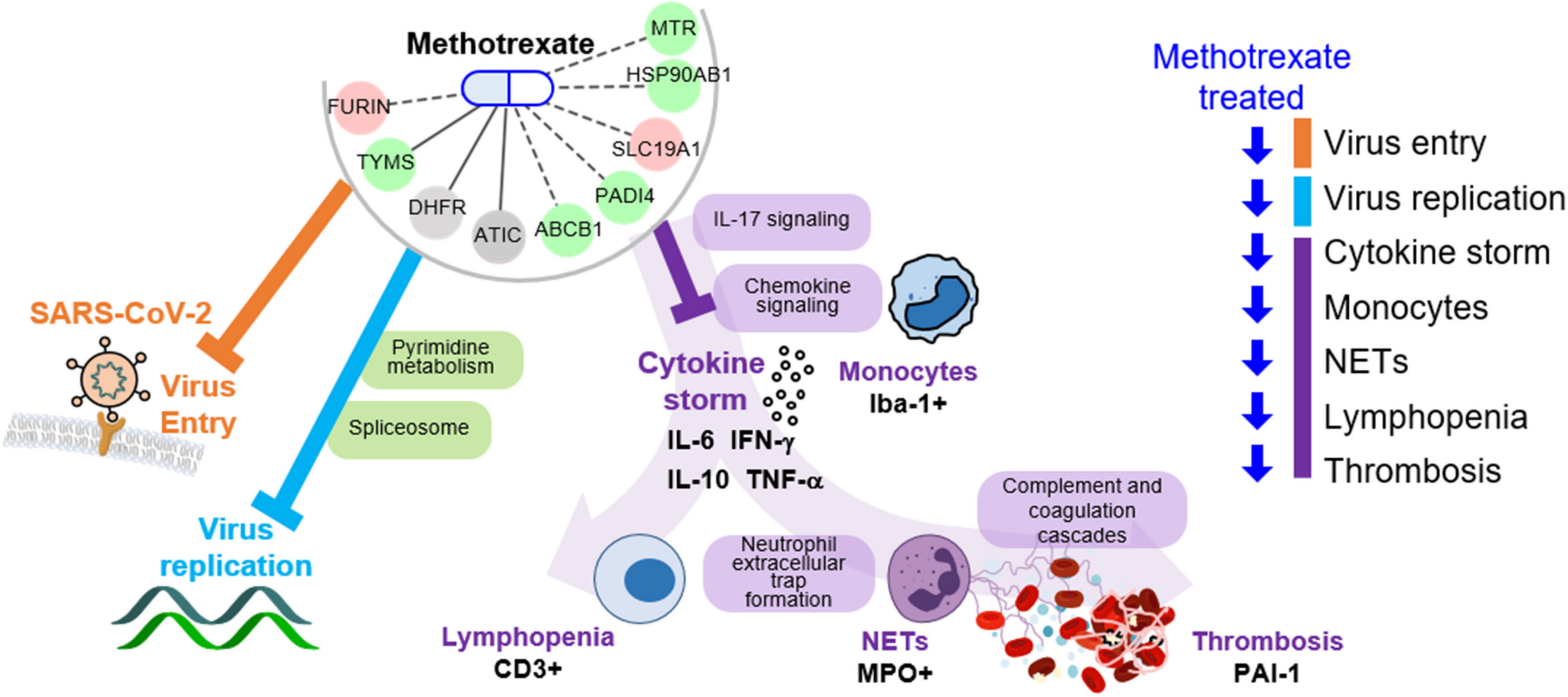
Schema of methotrexate function in COVID-19. Methotrexate inhibits SARS-CoV-2 entry (*via* target FURIN; orange) and regulates pyrimidine metabolism and spliceosome pathways (*via* TYMS, DHFR and ATIC; light blue) to inhibit virus replication. Methotrexate suppresses COVID-19 immunopathologies (purple), including monocyte-driven cytokine storm (by regulating IL-17 and chemokine signaling), NETs formation, thrombosis (by regulating complement and coagulation cascades) and lymphopenia. The methotrexate targets expression in treated hamsters is shown, and the validated biomarkers are shown by black labels.

The key advantage of our method is the identification of both multi-target drugs and their mechanisms by inferring COVID-19-specific associations of genes, drugs and protein-drug interactions to discover FDA-approved drugs. Here, we identified methotrexate as a multi-target drug: 1) inhibits SARS-CoV-2 entry, 2) reduces virus replication (*via* pyrimidine metabolism), and 3) diminishes the inflammatory monocyte-driven cytokine storm (*via* IL-17 signaling) to reduce NETs formation associated thrombosis (*via* complement and coagulation cascades) and lymphopenia (*via* T cell receptor signaling) (**Figure 6**). Importantly, these predictions were validated in a hamster model by lung omics and biomarker assays. These data indicate that our approach is indeed a useful tool for identifying multi-target candidates for further repurposing development for human diseases.

Since the outbreak of COVID-19 pandemic, scientists worldwide are racing to find effective COVID-19 treatments including drug repurposing (45, 46). However, less than 1% of clinical trials registered (~4/200) showed significant therapeutic effects. Thus, good activities *in vitro* does not warrant clinical success as shown in the case of HCQ (4). In this regard, although some previous reports indicated methotrexate’s *in vitro* activity (13, 47) and proposed for COVID-19 treatment (14) and even viral disease (48). However, it’s *in vivo* efficacy remained to be tested. Our work reports several unique and novel findings. Firstly, we provided a powerful strategy with a scoring function to discover drugs and their multiple targets, for complex diseases such as COVID-19 with multiple symptoms (*e.g.*, cytokine storm and thrombosis). For instance, here we discovered methotrexate inhibition of a novel target furin involved in SARS-CoV-2 entry. Secondly, our work is the first *in vivo* study to quantify methotrexate effects in SARS-CoV-2 lung infection, hyper-inflammation and histopathological evaluation, which were not previously explored. Thirdly, using lung transcriptomics we reveal the systemic effects of methotrexate to predict its mechanisms in COVID-19 (*e.g.*, innate and adaptive immunity, thrombosis) thus enabling further clinical evaluation.

The mechanism by which methotrexate inhibits SARS-CoV-2 infection deserves further consideration in the face of evolving virus variants. SARS-CoV-2 mutates quickly and at least 4 major variants appeared since the outbreak (22), which jeopardize the effectiveness of vaccines. Breakthrough infections have also been reported in vaccinated individuals. Drugs that directly target SARS-CoV-2 proteins could also be affected from the virus mutations. Therefore, devising better treatment strategies and drugs that remain unaffected by the viral mutations are needed to protect humans. Methotrexate inhibits multiple host proteins for which genes are stable and preserved. This feature is clearly advantageous in that efficacy is unlikely to diminish when treating different SARS-CoV-2 variants. Indeed, we showed methotrexate strongly inhibited all the four variants of concern in the current study. On the other hand, methotrexate could be combined with drugs that target directly SARS-CoV-2 virus to synergistically treat COVID-19. Nevertheless, these hypotheses should be addressed by treating patient populations in future clinical studies.

The advantages of methotrexate in treating COVID-19 are summarized as follows: (a) methotrexate achieves true multi-targeting in COVID-19, by inhibiting multiple targets from diverse protein families like proteases (furin), reductases (DHFR), synthases (TYMS) and transformylase (ATIC). This finding is different from the case of baricitinib that inhibits protein kinases only. Here, methotrexate’s additional targeting on virus entry is a new discovery. (b) Methotrexate mitigates broader clinical COVID-19 symptoms like NETosis, inflammation and thrombosis, critical for patient survival. (c) Methotrexate is a remarkable drug for COVID-19 due to the benefits like good efficacy and safety, options for oral or parenteral route and currently unrivalled cost-effectiveness. Additionally, as the burden of COVID-19 pandemic on people’s lives and health is specifically worse in developing countries, an immediate access to easily accessible and affordable anti-COVID-19 drug such as methotrexate, can be a game changer for patients in these regions. (d) Methotrexate showed systemic and cellular anti-COVID-19 effects often comparable, sometimes better than the standard drug remdesivir. As methotrexate strongly inhibits SARS-CoV-2 replication and reduces inflammation, it could be effective in treating patients with mild to moderate illness, prevent disease progression to severe illness and death (49). Furthermore, based on clinical use of methotrexate in other diseases (such as RA), we believe that methotrexate could be safely used in pediatric (50) as well as aging patients (51). with no age-bias. Also, there were no significant differences in methotrexate prescriptions based on gender as discussed in Giusti et al. (52), thus no gender-bias. However, use of methotrexate in COVID-19 patients with other concomitant disease conditions needs careful evaluation, pointing to future studies considering comorbidities. For instance, methotrexate use in patients with coronary artery disease (CAD) could be particularly beneficial, due to reduced risk of cardiovascular diseases events (observed in methotrexate treated RA patients with CAD) (53). In contrast, methotrexate administration may induce adverse effects such as gastrointestinal complications in patients with renal dysfunction (54).

The approach, combining Homopharma and HiSBiN, in the current study, however has some limitations and challenges. First, the predicted drug-target relationships still need to be experimentally validated. Furthermore, the prediction performance although has been verified by Set_E and Set_C (**Figures S1E, F**), needs more systematic validation to figure out its strengths and weaknesses. However, as there are still no golden positive COVID-19 drug sets, future successful clinical drugs will help to achieve this. Thirdly, owing to the limited structural data, we predicted potential targets for a drug using both structure and sequence similarities, thus our approach may lose the targets having similar binding pockets but with low sequence similarity. Finally, our predictions do not consider host-pathogen interactions because the pathway databases (such as KEGG) are often incomplete, and the lung omics data used for DEGs contains host genes only. These challenges could be resolved in the future, leading to an improvised method.

Hamsters has become one of the standard animal models to study SARS-CoV-2 due to its high susceptibility. However, the disadvantages are that it is non-lethal, the lack of anti-hamster cytokine antibodies and pharmacokinetic data, which limits the extent of experimental analysis. The mechanisms of methotrexate in the hamster omics analyses, provide interesting clues connecting inhibition of molecular targets to systemic effects. However, for these large-scale high throughput approaches, it is challenging to reveal strong and detailed evidences for each of the mechanisms. Some supporting evidences that could help address this challenge are discussed as follows. Methotrexate’s direct inhibition of its multiple molecular target proteins such as DHFR, TYMS and ATIC suppresses nucleotide biosynthesis, and promotes adenosine release and ROS production processes in the cell (27, 38). In severe COVID-19 cases, the deregulation of innate immune system and abnormal activation of neutrophils and monocyte/macrophage has been reported in several previous studies (32, 55, 56). For instance, the methotrexate-induced adenosine release *via* ATIC is supposedly involved in reduction of neutrophils (confirmed by IHC of MPO^+^ cells) and formation of NETs observed in methotrexate-treated hamsters, contributing to its anti-inflammatory effects. Methotrexate-induced adenosine release could also lead to inhibition of JAK-STAT signaling (*via* a canonical ISGF3 pathway that induces Mx1 (57)) leading to decreased Mx1 (Mx1^+^ cells in **Figure 4D**). In another example, methotrexate decreased the activated macrophages (Iba-1^+^ in **Figure 4**) in SARS-CoV-2-infected hamsters, which could be similar to its inhibition of macrophage activation and conditioning for decreased pro-inflammatory cytokines like TNF-α and IL-6, observed in RA (58). These and many other anti-COVID-19 mechanisms of methotrexate can be further validated by detailed future studies.

In conclusion, our systematic drug repurposing approach is able to discover disease-specific multi-target drugs, pharmaceutical targets and regulated pathways. Additionally, methotrexate showed remarkable anti-COVID-19 efficacy by modulating multiple host genes and pathways to treat SARS-CoV-2-induced lung damage and inflammation. Thus, methotrexate is a promising drug for future clinical evaluation in COVID-19 patients.

## Material and methods

### Study approval

Experiments using infectious SARS-CoV-2 have been approved by the Institutional Biosafety Committee (IBC) and were performed in the high biocontainment BSL3 and ABSL3 facilities of the Institute of Preventive Medicine (IPM), National Defense Medical Center (NDMC), which are approved for SARS-CoV-2-related studies by the Taiwan Centers for Disease Control, under license D1–109–0030#1123 and D1–0031#1124. All animal experimentation was reviewed and approved by IPM’s Institutional Animal Care and Use Committee (IACUC) under permits AN-109–31 and AN-110–08, and was performed according to the standard operating procedures of the Animal Biosafety Level 3 facilities.

### Animal models

In this work, we designed our hamster experiments with n=3 based on literature on COVID-19 animal models (59, 60) which used n=3 for hamster experiments, and also due to limited capacity of our ABSL3 lab. Twelve male Syrian golden hamsters, aged 7−9 weeks old were obtained from the National Laboratory Animal Center, Taipei, Taiwan. The animals were kept in specific-pathogen-free (SPF) housing and were acclimatized at the ABSL-3 facility for 3 days before the experiments. Hamsters were randomly allocated to study groups for antiviral evaluation. About 100 μL of SARS-CoV-2 (1×10^4^ PFU) was intranasally instilled under intraperitoneal anesthesia with Zoletil50 (4 mg/kg)/Xylazine (0.5 mg/kg) at day 0, and the mock-infected hamsters were challenged with 100 μL of PBS. Body weight and clinical signs of the disease in the hamsters were monitored daily during the study as a measure of disease progression.

For the drug treatment groups, on the day of infection, a loading dose of 2 mg/kg of methotrexate or 5 mg/kg of remdesivir was given; on 1^st^ to 3^rd^ day post-infection (dpi), a daily dose of 2 mg/kg of methotrexate or 2.5 mg/kg of remdesivir was given (**Figure 3A**). Clinically, methotrexate (MTX) is used in high doses to treat various malignancies such as acute lymphoblastic leukemia and lymphoma (61). However, it is also used in low doses to effectively treat inflammatory diseases such as rheumatoid arthritis and Crohn’s disease (62, 63). The methotrexate doses in animal experiments ranges from 2mg/kg to 200mg/kg (64, 65). For the dose in our experiments, due to lack of hamster pharmacokinetics we used the mice pharmacokinetics by Lobo ED’s lab (66), and to avoid methotrexate-induced toxicity (67) to consider a lower test dose of 2mg/kg/day for short-term treatment. On the 4^th^ dpi, the blood was harvested for hematological analysis. All hamsters were euthanized, and the lungs were collected and divided into 3 parts: for viral yield measurement by qRT-PCR and plaques, for RNA extraction for desired gene-cytokine-chemokine expression profiles, and for histological examinations.

### Gene expression analyses for public SARS-CoV datasets

Public omics datasets GSE5972 and GSE1739 of SARS-CoV-infected patients’ peripheral blood samples were collected from the NCBI Gene Expression Omnibus (GEO) (68). GSE5972 included different stages of the patients, precrisis, crisis and discharge (69), so we separated them into two datasets (*i.e.,* GSE5972: precrisis and GSE5972: crisis) considering distinct phases of illness by samples taken at different days after disease onset. For GSE1739, we analyzed 14 samples of peripheral blood mononuclear cells (PBMCs) from 4 control and 10 infected patients (70). Each sample comprised 8,793 probes using the Affymetrix human genome (HG)-Focus microchip. For GSE5972 which comprises 19,200 probes and 64 samples, 10 control, 26 precrisis and 28 crisis patients were considered (69). The gene expression levels were measured on the UHNMAC Homo sapiens 19K Hu19Kv8 microarray platform. For each data set, the DEGs were selected between normal and post-infection samples, based on fold change (FC) ≥ 1.5 and p value< 0.05, calculated using Student’s *t*-test through the GEO2R analysis tool (https://www.ncbi.nlm.nih.gov/geo/geo2r/), to derive 2,516 DEGs from the union of the three public GEO datasets (**Figure S2A** and **Supplementary Data 1**) (68).

### Analysis of the SARS-CoV-2 RNA-seq datasets

For the RNA-seq data from six hamster samples, the quality of reads was examined using FastQC (https://www.bioinformatics.babraham.ac.uk/projects/fastqc/). The reads were aligned to the Syrian hamster (*Mesocricetus auratus*) genome MesAur1.0 (https://www.ncbi.nlm.nih.gov/assembly/GCF_000349665.1/) using HISAT2 (details on mapped reads in **Table S2**) (71). The average mapping rate of all reads was 88.82% and the mapped reads from HISAT2 were assembled into transcripts using StringTie (72). Then, the expression profiles of all transcripts were evaluated and computed on fragments per kilobase of transcript per million fragments mapped (FPKM) using StringTie and the Ballgown package in R (73). For the gene expression profiles generated using 58,830 probes for six samples, log2 transformation was performed to measure the FC of mRNA expression. Then, we selected DEGs between normal and SARS-CoV-2 infected hamster samples based on FC ≥ 2 (**Figure S2D**) by using the limma R package. To evaluate the RNA-seq samples, principal component analysis (PCA) was performed (**Figure S2E**) to analyze the gene expression of RNA-seq data by using WebMeV (Multiple Experiment Viewer) (74), in which the three sample groups were separated, and samples of the same group were clustered together. Moreover, methotrexate-treated samples were closer to mock samples than that of untreated ones.

For comparing the SARS-CoV and SARS-CoV-2 patient profiles, we also collected the GSE163151 dataset, which includes control and SARS-CoV-2 samples from human nasopharyngeal tissue (93 control and 138 SARS-CoV-2 samples) and whole blood (20 control and 9 SARS-CoV-2 samples). The RNA-seq data comprised 26,485 probes and the data was sequenced using an Illumina NovaSeq 6000. The normalized data were analyzed using the DESeq package in R and the DEGs with FC ≥ 2 and p value < 0.05 were selected (**Figure S2B**). Hierarchical clustering of public SARS-CoV and SARS-CoV-2 datasets based on their DEG enrichment in pathways was performed (**Figure S2C**) and the results showed clustering of upregulated gene pathways of SARS-CoV-2 with SARS-CoV and the same for downregulated gene pathways, indicating similar affected pathways and disease states.

### Drugs, inhibitors, targets and drug–target pairs

We aim to repurpose approved drugs for facilitate the treatment of COVID-19. To predict the approved drugs for COVID-19, we collected three drug-target sets. First, we collected FDA-approved drugs from the DrugBank database (75) (v5.1.7) according to the following criteria: (i) annotated “approved” in the “Groups” column and “small molecule” in the “Type” column; and (ii) the number of heavy atoms (HA) of the drug is 7≤ HA≤ 125 to obtain 2,303 drugs (Set_D). Then, we collected their 2,363 druggable targets (Set_DT) from DrugBank to get 9,130 drug-target pairs. To infer similar approved drugs of DEG inhibitors using Homopharma, we secondly collected the inhibitors with bioassay data from BindingDB database (76) (version 2020m10) satisfying three criteria: (i) its binding affinities, including one of Ki, Kd or EC_50_ values ≤10 μM, (ii) its targets marked as “reviewed” in UniProt database, and (iii) one of its targets in 2,516 SARS-CoV infected DEGs. In total from the BindingDB, we retrieved 147,754 inhibitors (Set_I) of DEGs and 162,155 inhibitor-DEG pairs. Third, we collected 158 drug-target pairs by text mining in COVID-19 related literature. As of June 2021, over 140,000 studied had “COVID-19” or “SARS-CoV-2” in the title/abstract. Furthermore, to evaluate the performance, we additionally collected two types of reference COVID-19 inhibitors. We collected 77 drugs that had positive outcomes in Vero E6 cells, 37 of which had a strong effect (46), denoted Set_E. Then, we selected 58 drugs (Set_C) from ClinicalTrials.gov (until 2021/06) with two criteria: i) the clinical status was “completed” and ii) the drug was a small compound (*i.e.,* 7 ≤*HA ≤* 125 recorded in DrugBank.

### Homopharma and docking analysis

For drug repurposing, we previously proposed the concept of Homopharma (15), in which compounds with similar topologies often bind to similar proteins having similar pockets in protein-compound interfaces (**Figures S1B, E, H**). For a DEG-drug/inhibitor pair (complex), we predicted the DEG as a potential drug target if, its inhibitor is similar to FDA-approved drugs or its homologous proteins are recorded in DrugBank. We predicted similar drugs of a DEG drug/inhibitor by searching the FDA-approved drugs database by their similarity score (*C.S.> 0.8*) calculated using Tanimoto similarity with 214 compound features (204 checkmol and 10 atom composition features) (77). We also inferred the homologous drug target of a DEG with the following criteria (1): significant sequence similarity BLASTP *E*-values ≤ 10^-10^ (2); aligned sequence coverage > 70%; and (3) sequence identity (*S.I.*) > 30%. Finally, if the pair has a structure complex, we used the in-house tool iGEMDOCK to dock the drug candidates into the target protein (or) aligned the predicted homologous drug target and drug candidates to the target protein and ligand in the complex respectively. In addition, for these docked or aligned proteins and compounds, we explored conserved interactions formed by conserved residues and similar functional groups to compute their binding scores and potential repurposing.

To further investigate the binding mechanisms of the drugs and targets, we applied our previous tools (*e.g.*, iGEMDOCK (78)) to simulate the binding mechanisms. For the methotrexate case, structures of furin binding site using reference peptide-mimic (ARG-ARG-ARG-LYS-ARG-00S) bound structure 6HZD was downloaded from Protein Data Bank (PDB) and the binding site was extracted radius around the ligand within ≤ 9 Å. We docked methotrexate and folic acid into the extracted binding cavities by iGEMDOCK, using the docking parameters 1,000 population size, 80 generations and 100 poses. For each pose, the total protein-ligand interaction energies including electrostatic, hydrogen bond, and van der Waals interactions were calculated. The docked poses were clustered based on similarity in the interaction profile and compound binding at the active site subpockets. The optimal poses for methotrexate and folic acid from clusters were chosen, if they (i) occupied the key binding subpockets, (ii) had a pose similar to the RRARS peptide, and (iii) interacted with one or more catalytic residues. We used the same procedure for the binding mechanism of pimozide and valsartan in their respective target proteins (**Figures S3D–I**).

### Target–pathway pairs by hierarchical systems biology network

To evaluate how DEGs and drug targets are involved in pathways during SARS-CoV-2 infection, we first collected 342 human pathways containing 7,806 proteins (genes) from the Kyoto Encyclopedia of Genes and Genomes (KEGG) database (79). We then modified the hierarchical systems biology model (HiSBiM) (16) from our previous work to measure the involvement of drug targets in the KEGG pathways, including DEG identification, pathway enrichment, and subsystem contribution by ‘hierarchical systems biology network (HiSBiN)’.

The DEGs for COVID-19 were identified based on fold change and p value between normal and SARS-CoV (or SARS-CoV-2) infection samples. To yield KEGG pathway enrichment of the DEGs, the p value of hypergeometric distribution was calculated as follows:


(1) $$p=\sum_{i=x}^{n}\frac{{(}_{i}^{M}){(}_{\text{n-i}}^{\text{N-M}})}{{(}_{n}^{N})}$$

where *N* is the number of all genes in the KEGG database, *M* is the number of DEGs, *n* is the number of genes and *i* is the number of DEGs in a specific KEGG pathway.

To understand how each DEG is involved in KEGG subsystems and pathways, we defined the SHiSBiN of a gene as follows:


(2) $$S_{HiSBiN}=\frac{Z_{Subs}+Z_{path}}{2}\;$$

$$Z_{Subs}=\frac{\sum_{i=1}^{m}Z_{i}}{\sqrt{m}}\;;\;Z_{Path}=\frac{\sum_{j=1}^{n}Z_{j}}{\sqrt{n}}$$

where *Z_Subs_* and Z*_Path_* are the meta-*z*-scores of the subsystems and pathways in which a gene is involved; Z*_i_* and Z*_j_* are the *z*-scores of subsystem *i* and pathway *j*, respectively; and *m* and *n* are numbers of a DEG-involved subsystems and pathways, respectively. The *z*-score of each pathway was the p value transformation using a standard normal distribution

### Identifying disease therapeutic targets by *S_DisG_*

To systematically infer the disease therapeutic targets, we developed a disease gene score (*S*_*DisG*_) and scored the DEGs. The *S*_*DisG*_*i*__ score (**Figure S5A**) of a DEG(*i*) considers (a) the gene expression significance p value (*S*_*PV*_*i*__) in disease and corresponding normal samples; (b) druggability (*S*_*Druggability*_*i*__) measuring the number of interacting drugs; and (c) HiSBiN derived significant gene involvement in disease pathways and subsystems (*S*_*HiSBiN*_*i*__). The score is computed as follows:


(3) $$S_{DisG_{i}}=w_{PV}\times S_{PV_{i}}+w_{D}\times S_{Druggabiliy_{i}}+w_{p}\times S_{HiSBiN_{i}}$$

where *i* is the DEG(*i*) and *w*_*PV*_, *w*_*D*_ and *w*_*p*_ are the weights of *S*_*PV*_*i*__, *S*_*Druggabiliy*_*i*__ *and* *S*_*HiSBiN*_*i*__,  respectively. Based on the systematic analysis, *w*_*PV*_, *w*_*D*_ and *w*_*p*_ are set to 0.5, 2, and 1, respectively.

For deriving (a), we consider the importance of genes in disease using *S*_*PV*_*i*__ , which is the maximum p value of target *i* gene expression (calculated by the GEO2R analysis tool) in the two datasets (GSE5972: precrisis, crisis and GSE1739) computed as follows:


$$S_{PV_{i}}=\text{max}\left(-log_{10}\left(p\;value_{i}^{s}\right)\right)$$

where *s* is the number of datasets.

For (b), *S*_*Druggability*_*i*__ measures the druggability of a DEG(*i*), based on the number of targeting drugs derived from Homopharma, computed as follows:


$$S_{Druggability_{i}}=a+\;\sum_{j=1}^{\beta }S_{hp_{j}}\times \beta$$

where α is the number of reported drugs for DEG(*i*), $S_{hp_{j}}$ is the Homopharma score of drug *j* (which is *C*.*S*. when predicted by compound similarity and *S*.*I*. when predicted by protein homology) and *β* is the number of predicted drugs of DEG(*i*). For example, DEG *PADI4* is a methotrexate target and is predicted to be targeted by 4 drugs (*α* = 4) (*e.g.*, azithromycin, tetracycline, streptomycin, and citrulline), as reported in drug–target pair data (*i.e.*, DrugBank), and by 42 drugs, as predicted from Homopharma (*β* = 42) (**Figure S5B**).

For (c), to evaluate how DEGs and drug targets are involved in pathways, we first identified 342 human pathways containing 7,806 proteins/genes in the KEGG database and modified the hierarchical systems biology model (HiSBiM) from our previous work (16) to measure the involvement of drug targets in these KEGG pathways, including DEG identification, pathway enrichment, and subsystem contribution, obtaining the “hierarchical systems biology network (HiSBiN)”. To determine the KEGG pathway enrichment of the DEGs, the p value of the hypergeometric distribution was calculated by Equation (2). A detailed example of the gene *PADI4* is shown in **Figure S5C** and **Supplementary Data 2**.

To evaluate the performance of identifying disease therapeutic targets for COVID-19, we employed the precision and recall to systematically evaluate the disease gene score (*S*_*DisG*_*i*__). Precision and recall are defined as TP/(TP+FP) and TP/(TP+FN) respectively, where TP, FP, and FN are the numbers of true-positive, false-positive, and false-negative cases. The 325 positive druggable COVID-19 genes (**Supplementary Data 3**) those overlapping between the 1,544 COVID-19 genes from DisGeNET (80) (No. of PMIDs ≥ 2) and the current 2,516 COVID-19 DEGs.

### Systematic drug score for identifying multi-target drugs for COVID-19

To predict multiple target drug candidates for COVID-19, we developed a systematic drug score *S*_*drug*_ that was calculated as follows:


$$S_{drug}=\sum_{i=1}^{T}(DT_{i})/\sqrt{T}$$

$$DT_{i}=w(w_{pv}PV_{i}+w_{path}S_{HiSBiNi})$$

$$w=\{\begin{array}{c} 1\;\;\;if\;\left(drug-target\;i\right)\;recorded\;in\;DrugBank\;\\ 0.5\;\;C.S.\;if\;target\;i\;predicted\;by\;compound\;similar\;\\ 0.5\;\;S.I.\;if\;target\;i\;predicted\;by\;protein\;homologous \end{array}$$

where *T* is the target number of a drug; *DT_i_* is the score of target protein *i*; *w_pv_* (= 0.7) and *w_path_* (= 0.3) are the weights of *PV* and *S*_*HiSBiN*_ defined in Equation (2). *PV_i_* is the average p value of target *i* of gene expression in three SARS-CoV GEO datasets (GSE5972: precrisis, GSE5972: crisis and GSE1739) and was computed as follows:


$$PV_{i}=\sum_{k=1}^{3}-log_{10}\left(p\;value_{i}^{k}\right)/k$$

The p value was calculated by the GEO2R analysis tool, *k* was number of datasets, and then *PV_i_* was normalized from 0 to 1. In **Figure S1D**, we used an example drug pimozide and described the detailed computing steps for all scoring terms. Finally, we computed the *S_drug_* values of 2,303 FDA-approved drugs.

We combining Homopharma and HiSBiN was applied to discover multi-target anti-COVID-19 drugs (see **Figure S1**). We first investigated the genes associated with disease, DEGs of three omics datasets from GSE5972 and GSE1739, including precrisis and crisis of human SARS-CoV-infected samples (**Figures S1A**, **S2A**). Second, we predicted potential approved drug candidates for COVID-19 using Homopharma, HiSBiN, and multi-target drug scores (**Figure S1B**). Third, we clustered these predicted drug candidates and selected the representative drugs for bioassay evaluation (**Figure S1C**). Additionally, we also evaluated the reliability of our method for COVID-19 drug predictions based on the two reference sets, Set_E and Set_C (**Figures S1E, F**).

### Performance evaluation of the integrated approach

We evaluated the reliability of our method to predict multi-target drugs for COVID-19, utilizing two independent reference sets collected, Set_E and Set_C (described in the previous section), by quantifying the method’s predictive power of the drug sets. The performance parameter recall is defined as TP/(TP+FN), where TP, FP, and FN are the numbers of true-positive, false-positive, and false-negative cases, respectively. The percentile rank is defined as [*M/D*] × 100, where *M* is the number of ranked drugs at percentile *X*, *D* is the total number of ranked drugs (**Figures S1E, F**). From the results, we found that our approach improved the prediction of ranked drugs from these drug sets. The prediction with only Homopharma (1,575 drugs) showed improved performance compared with that without Homopharma and HiSBiN (619 drugs). The prediction with Homopharma with added HiSBiN, showed improvement (1,588 drugs) and had the best performance. Furthermore, the prediction applied in the current case of multi-targeting (with target *T* = 4), showed similar good performance (1,592 drugs). Additionally, for the Set_E, the integration (with both Homopharma and HiSBiN) showed the best recall of 0.33 at the percentile rank of 25%, while for Set_C, it showed the best recall of 0.3 at the percentile rank of 25%.

### Cells and viruses

Vero E6 cells (an African green monkey kidney cell line, ATCC CRL-1586) were maintained in high glucose DMEM (HyClone), supplemented with 10% fetal bovine serum (FBS, HyClone) and an antibiotic-antimycotic (Gibco) in a humidified atmosphere of 37°C and 5% CO2. The SARS-CoV-2 WA strain used in this study was kindly provided by Chang Gung Memorial Hospital, and the four VOCs namely B.1.1.7, B.1.351, P.1, and B.1.617.2, all of which were kindly provided by Taiwan Centers for Disease Control, Ministry of Health and Welfare and propagated using Vero E6 cells supplemented with 2% FBS. Passage 2 virus was used for the all the studies described here. Viral stocks were free of contamination and confirmed to be identical to the initial deposited GenBank sequence (MT370517.1). The viral titers were determined by plaque assay followed by storage of aliquots at −80°C until further use in the experiments.

### Cytotoxicity, antiviral cytopathic effect and immunofluorescence assays

The cytotoxicity and antiviral activity of methotrexate were evaluated by protocols similar to those described in our previous work (77). In the cytotoxicity assay, Vero E6 cells (1×10^4^ cells/well) were seeded in a 96-well culture plate overnight. The cells were then treated with increasing concentrations of methotrexate with or without the control for 72 h. Media were removed after incubation and 50 µL of the tetrazolium salt (3-[4,5-dimethylthiazol-2-yl]-2,5-diphenyl-tetrazolium bromide) MTT solution (5 mg/ml in phosphate-buffered saline (PBS) was added to the cells for 4 h to allow MTT formazan formation. After removing the medium, 100 µL of dimethyl sulfoxide (DMSO) was added to dissolve the formazan crystals. The absorbance of each well was measured at a 590-nm wavelength with an EMax precision microplate reader (Molecular Devices, USA). The cytotoxic concentration (CC_50_) was calculated as the concentration of the compound that decreased cell viability by 50%, as compared to cells treated with DMSO alone.

The antiviral activity of a drug was evaluated by measuring the inhibition of virus-induced cytopathic effect (CPE). Vero E6 cells (1×10^4^ cells/well) were seeded in 96-well culture plates for approximately 24 hours. When the cell cultures were confluent, the culture medium was removed, and 100 μl of media (DMEM with 2% FBS) containing 10 median tissue culture infectious doses (TCID_50_) of virus and serial 2-fold dilutions of drugs were added simultaneously. For the virus-only control and cell-only control, the virus suspension and working medium (DMEM with 2% FBS) without drugs were added and incubated (48 h for SARS-CoV-2 WA, 72 h for the VOCs). The concentration of the compound that reduced 50% of CPE to that induced by the virus-only control was defined as the 50% effective concentration (EC_50_). Both the CC_50_ and EC_50_ curves were plotted and calculated by Prism 8.0 software (GraphPad Software Inc.).

For the immunofluorescence assays, antigen expression in SARS-CoV-2-infected Vero E6 cells was detected with a mouse monoclonal antibody against SARS-CoV-2 spike protein (Genetex, GTX632604) followed by a DyLight4-conjugated secondary antibody (Abcam). Cell nuclei were labeled with the nucleic acid stain 4,6-diamidino-2-phenylindole (DAPI) (Sigma).

### Pseudovirus neutralization assay

To produce the SARS-CoV-2 spike pseudovirus, the following three plasmids were co-transfected into Lenti-X 293T cells (Takara Bio USA): pLAS2w.Nluc-T2A-RFP-C. Ppuro, pCMVdeltaR8.91, and pcDNA3.1–2019-nCoV-S-d18. The latter two plasmids were obtained from the RNAicore facility of Academia Sinica, Taipei, Taiwan. The reporter plasmid pLAS2w.Nluc-T2A-RFP-C. Ppuro was cloned by insertion of the Nluc (NanoLuc, from Promega)-Gly-Ser-Gly-T2A sequence upstream of the RFP coding sequence in a lentiviral vector (pLAS2w.RFP-C. Ppuro, acquired from the RNAicore, Academia Sinica) by In-Fusion cloning (Takara). After transfection, cell culture supernatants were collected as viral stock of SARS-CoV-2-spike pseudovirus. For the pseudovirus neutralization assay, HeLa cells stably expressing SmBiT-hACE2 (81) were cultured in 96-well white plates at a density of 3×10^5^ cells per well one day before viral infection. Cells were treated with DMSO control or methotrexate and simultaneously infected with SARS-CoV-2-spike pseudovirus for 6 hr. The infected cells were washed three times with phosphate-buffered saline and incubated for an additional 18 hr. A Nano-GLo live-cell assay was used to measure intracellular NanoLuc luciferase activity (Promega). The luminescence signal was recorded immediately by a luminescent microplate reader (BioTek Synergy HTX) at 37 °C with a time-lapse kinetics program of 2 min intervals for 1 hr. To calculate the percentage of neutralization, luminescent data from the time point showing the highest signal in the negative control sample were chosen for downstream calculation. The percentage of neutralization (%) was calculated by 1- ((luminescence signal of the test sample)/(luminescence signal of negative control sample))*100, and the results were plotted as graphs.

### Time-of-addition assay

A time-of-addition assay was performed to investigate the stage of the SARS-CoV-2 life cycle affected by the inhibitor compounds. Vero E6 cells were seeded in 6-well plates (8×10^5^ cells per well). The cells were infected with SARS-CoV-2 (MOI of 0.01) and then incubated for 1 h. The viral inoculum was then removed and the cells were washed twice with PBS. In the entry group, drugs were added together with the virus inoculation at 0 hours post infection (hpi), followed by drug removal at 1 hpi. In the post-entry group, drugs were added 1 h after SARS-CoV-2 inoculation, followed by incubation at 37 °C in 5% CO_2_ until 24 hpi. The group treated with the drug for the entire experiment course of the infection was used as a positive control and the group treated with DMSO was included as a negative control. At 24 hpi, the cell culture supernatant was collected for viral yield using qRT-PCR quantification. The % inhibition was calculated by normalizing to the DMSO-only group for each time group and graphs were plotted by Prism 8.0 software (GraphPad Software Inc.).

### Rescue of SARS-CoV-2 replication after methotrexate treatment by folinic acid

A monolayer of Vero E6 cells (1×10^4^ cells/well) was prepared in a 96-well cell culture plate. Vero cells were then infected with SARS-CoV-2 at 100 TCID_50_ and simultaneously treated with 10 μM methotrexate, 50 μM folinic acid or a combination of 10 μM methotrexate with 50 μM folinic acid (Sigma-Aldrich, USA, #F7878), followed by 72 h incubation at 37°C with 5% CO_2_. The cell viability was determined with an MTT assay. For western blotting, a monolayer of Vero E6 cells (8×10^5^ cells/well) was prepared in a 6-well cell culture plate. Vero cells were then infected with SARS-CoV-2 at an MOI of 0.01, with 10 μM methotrexate, 50 μM folinic acid or a combination of 10 μM methotrexate with 50 μM folinic acid. The cells were further incubated for 24 h at 37°C and 5% CO_2_. After incubation, the cell lysate was collected to measure viral spike protein expression by western blot.

### Viral titers, viral RNA extraction and quantitative PCR in hamster lungs

To determine virus titer, virus isolation was performed on the lungs by homogenizing the tissues in 1 mL DMEM using a bead disruption (Precellys). About 200 μL of a 1:10 serially diluted lung lysate was inoculated into Vero E6 cells in a 24-well plate. One hour after inoculation of cells, the inoculum was removed and replaced with 1.4% methylcellulose in DMEM (HyClone) supplemented with 2% fetal bovine serum. Three days after incubation, the culture medium was removed, the cells were fixed with 10% (v/v) formalin and then stained with 0.5% (w/v) crystal violet and the plaques were counted. To measure virus yield in hamster lung homogenates by qRT-PCR, first, viral RNA was extracted and isolated in culture medium using a QIAamp Viral RNA Mini Kit (Qiagen) according to the manufacturer’s instructions. The lung tissue samples were homogenized using bead disruption (Precellys) in RLT buffer (RNeasy plus Mini Kit, Qiagen) and centrifuged (10,000 rpm, 5 min) to pellet cell debris, and RNA was collected according to the manufacturer’s protocol. For detection of viral RNA, 5 μl RNA was used in a one-step real-time RT-PCR against the E gene of SARS-CoV-2. RT-PCR was performed on an ABI *7500 Fast* Real-Time PCR System (Applied Biosystems) using the One-Step PrimeScript RT-PCR Kit (Takara) according to the manufacturer’s instructions. Dilutions of RNA standards quantified by droplet digital PCR were run in parallel and used to calculate gRNA copies with the E gene assay. Actin was used as the internal control for hamster mRNA analysis and the relative mRNA expression was calculated using the 2^-ΔΔCt^ method. The primer and probe sequences were listed in **Table S3**.

### Hamster lung histological and quantitative image analysis

The hamster lungs from different treatment groups were fixed in 4% paraformaldehyde (with two changes) for a minimum of 7 days and embedded in paraffin. Tissue sections (3 μm) were stained with hematoxylin and eosin (H&E) and examined blindly for lung damage by two board-certified veterinary pathologists. The severity of lesions was graded according to the methods described by Shackelford et al. (82). The degrees of lesions were graded histopathologically from zero to five depending on severity (0 = not present; 1 = minimal (< 1%); 2 = slight (1–25%); 3 = moderate (26–50%); 4 = moderately severe (51–75%); 5 = severe/high (76–100%)).

Additional IHC analyses were performed as follows: serial paraffin sections (3 µm) were deparaffinized by EZ prep (Ventana Medical Systems, Inc., Tucson, AZ, USA). The slides were incubated with antibodies against CD3 (Ventana Medical Systems, 790-4341), MPO (Santa Cruz, sc-365436 at 1:50), Iba-1 (Genetex, GTX100042 at 1:100), and Mx1 (Santa Cruz, sc-50509 at 1:50) for 32 min using the automated Ventana Benchmark XT (Ventana Medical Systems, Inc., Tucson, AZ, USA). Labeling was detected with the Ultraview DAB Detection Kit (Ventana Medical Systems, Inc., Tucson, AZ, USA) following the manufacturer’s protocol. All sections were counterstained with hematoxylin in Ventana reagent. Slides were immediately digitized with a MoticEasyscan pro Digital Slide Scanner (Motic) at ×40 (0.26 µm/pixel) with high precision (High precision autofocus) and the IHC staining was analyzed with Aperio ImageScope software (Aperio Technologies Inc.) using positive pixel count algorithm version 9.

### White blood cell population analysis

All hamster blood samples were collected, handled and processed in the same way at room temperature (approximately 22°C). Complete blood count (CBC) was carried out within two hours of sample collection using an automated hematology analyzer (XT-1800i, Sysmex Corporation, Kobe, Japan). All procedures were performed following the manufacturer’s instructions.

### PCR-based quantification of genes, cytokines and chemokines in hamster lungs

For the desired gene, cytokine and chemokine profiles, RNA was first isolated from hamster lung homogenate using an RNeasy Plus Mini kit (Qiagen) according to the manufacturer’s protocol. Diluted RNAs (1:5) were used for one-step qRT-PCR by using a QuantiFast SYBR Green RT-PCR Kit (Qiagen) on a 7500 Fast real-time PCR system (Applied Biosystems). Actin was used as the internal control, and the relative mRNA expression was calculated using the 2^-ΔΔCt^ method. The list of the primers is provided in **Table S3**.

### Quantification and statistical analysis

Statistics were performed by GraphPad Prism version 8 (GraphPad Software Inc.) noted with the appropriate tests outlined in the figure legends. p< 0.05 was considered statistically significant. Due to the low number of animals included in our study, p values ≤ 0.1 have been indicated in the graphs. Ranges of significance were graphically annotated as follows: p< 0.05; *p<0.01; **p< 0.001; ***p< 0.0001.

## Data availability statement

The data presented in the study are deposited in the GEO repository, accession number GSE179709, which is available at https://www.ncbi.nlm.nih.gov/geo/query/acc.cgi?acc=GSE179709.

## Ethics statement

Experiments using infectious SARS-CoV-2 have been approved by the Institutional Biosafety Committee (IBC) and were performed in the high biocontainment BSL3 and ABSL3 facilities of the Institute of Preventive Medicine (IPM), National Defense Medical Center (NDMC), which are approved for SARS-CoV-2-related studies by the Taiwan Centers for Disease Control, under license D1–109–0030#1123 and D1–0031#1124. All animal experimentation was reviewed and approved by IPM’s Institutional Animal Care and Use Committee (IACUC) under permits AN-109–31 and AN-110–08, and was performed according to the standard operating procedures of the Animal Biosafety Level 3 facilities.

## Author contributions

Investigation, Y-TC, Y-HC, NP, Y-Lu, Y-CH, J-YL, Y-CC, Y-WH, H-JY, N-YH, and J-MY. Methodology, Y-TC, Y-HC, NP, Y-Lu, Y-CH, T-NL, J-YL, Y-CC, Y-WH, H-PT, T-YC, S-CH, P-CL, Y-FC, W-CL, C-MY, H-LW, C-YL, H-LH, Y-Li, and J-MY. Formal analysis, Y-HC, NP, Y-Lu, Y-CH, T-NL, and J-MY. Resources, LW, C-HL, Y-JH and J-MY. Conceptualization, S-CT, C-HH, P-SH, Y-HL, Y-JH, and J-MY. Supervision, S-CT, LW, C-HL, Y-HL, Y-JH, and J-MY. Writing – original draft, Y-TC, Y-HC, NP, Y-Lu, Y-CH and J-MY. Writing – review and editing, S-CT, LW, J-WC, J-YW, Y-HL, Y-JH, and J-MY. All authors contributed to the article and approved the submitted version.
