# Supplementary material for: Methotrexate inhibition of SARS-CoV-2 entry, infection and inflammation revealed by bioinformatics approach and a hamster model
Source: Front Immunol. 2022 Dec 21;13:1080897. doi: 10.3389/fimmu.2022.1080897 (PMC9811668; doi:10.3389/fimmu.2022.1080897)
Supplement: Supplementary file 4 [file DataSheet_4.pdf]

SUPPLEMENTAL INFORMATION

Methotrexate inhibition of SARS-CoV-2 entry, infection and inflammation revealed by bioinformatics approach and a hamster model

Yun-Ti Chen, Yu-Hsiu Chang, Nikhil Pathak, Shey-Cherng Tzou, Yong-Chun Luo, Yen-Chao Hsu, Tian-Neng Li, Jung-Yu Lee, Yi-Cyun Chen, Yu-Wei Huang, Hsin-Ju Yang, Nung-Yu Hsu, Hui-Ping Tsai, Tein-Yao Chang, Shu-Chen Hsu, Ping-Cheng Liu, Yuan-Fan Chin, Wen-Chin Lin, Chuen-Mi Yang, Hsueh-Ling Wu, Chia-Ying Lee, Hui-Ling Hsu, Yi-Chun Liu, Jhih-Wei Chu, Lily Hui-Ching Wang, Jann-Yuan Wang, Chih-Heng Huang, Chi-Hung Lin, Po-Shiuan Hsieh, Yan-Hwa Wu Lee, Yi-Jen Hung, Jinn-Moon Yang

Table S1. **KEGG pathways and gene ontology-biological processes (GO-BPs) abbreviation mapped table, related to Figures 1 and 5**

KEGG pathways	
**Full name**	**Abbreviation**
Chemokine signaling pathway	Chemokine signaling
Viral protein interaction with cytokine and cytokine receptor	cytokine and cytokine receptor
TNF signaling pathway	TNF signaling
IL-17 signaling pathway	IL-17 signaling
Antigen processing and presentation	Antigen processing and presentation
Intestinal immune network for IgA production	Intestinal immune for IgA production
Toll-like receptor signaling pathway	Toll-like receptor signaling
NOD-like receptor signaling pathway	NOD-like receptor signaling
Leukocyte transendothelial migration	Leukocyte transendothelial migration
PI3K-Akt signaling pathway	PI3K-Akt signaling
Cytokine-cytokine receptor interaction	Cytokine-cytokine receptor interaction
Coronavirus disease - COVID-19	Coronavirus disease - COVID-19
Cytosolic DNA-sensing pathway	Cytosolic DNA-sensing
Natural killer cell mediated cytotoxicity	Natural killer cell mediated cytotoxicity
Jak-STAT signaling pathway	Jak-STAT signaling
RIG-I-like receptor signaling pathway	RIG-I-like receptor signaling
Platelet activation	Platelet activation
Complement and coagulation cascades	Complement and coagulation cascades
p53 signaling pathway	p53 signaling
Cell cycle	Cell cycle
DNA replication	DNA replication
Protein processing in endoplasmic reticulum	Protein processing in ER
Spliceosome	Spliceosome
Pyrimidine metabolism	Pyrimidine metabolism

RNA degradation	RNA degradation
Aminoacyl-tRNA biosynthesis	Aminoacyl-tRNA biosynthesis
Nucleotide excision repair	Nucleotide excision repair
Mismatch repair	Mismatch repair
B cell receptor signaling pathway	B cell receptor signaling
Sphingolipid signaling pathway	Sphingolipid signaling
Fc gamma R-mediated phagocytosis	Fc gamma R-mediated phagocytosis
VEGF signaling pathway	VEGF signaling
mTOR signaling pathway	mTOR signaling
GnRH signaling pathway	GnRH signaling
Prolactin signaling pathway	Prolactin signaling
ErbB signaling pathway	ErbB signaling
Inflammatory mediator regulation of TRP channels	Inflammatory mediator regulation
**GO-BPs**	
**Full name**	**Abbreviation**
cytokine-mediated signaling pathway	cytokine-mediated signaling…(GO:0019221)
response to cytokine	response to cytokine (GO:0034097)
cellular response to cytokine stimulus	cytokine stimulus to cytokine…(GO:0071345)
leukocyte migration	leukocyte migration (GO:0050900)
leukocyte chemotaxis	leukocyte chemotaxis (GO:0030595)
myeloid leukocyte migration	myeloid leukocyte migration (GO:0097529)
granulocyte migration	granulocyte migration (GO:0097530)
granulocyte chemotaxis	granulocyte chemotaxis (GO:0071621)
granulocyte activation	granulocyte activation (GO:0036230)
neutrophil chemotaxis	neutrophil chemotaxis (GO:0030593)
neutrophil migration	neutrophil migration (GO:1990266)
neutrophil activation	neutrophil activation (GO:0042119)
regulation of defense response to bacterium	regulation of defense response…(GO:1900424)

regulation of neutrophil mediated cytotoxicity	regulation of neutrophil mediated⋯(GO:0070948)
regulation of neutrophil mediated killing of symbiont cell	regulation of neutrophil⋯(GO:0070949)
neutrophil mediated cytotoxicity	neutrophil mediated cytotoxicity (GO:0070942)
neutrophil-mediated killing of symbiont cell	neutrophil-mediated killing cell⋯(GO:0070943)
neutrophil mediated immunity	neutrophil mediated immunity (GO:0002446)
lymphocyte migration	lymphocyte migration (GO:0072676)
helper T cell diapedesis	helper T cell diapedesis (GO:0035685)
T-helper 1 cell extravasation	Th1 cell extravasation (GO:0035687)
T-helper 1 cell diapedesis	Th1 cell diapedesis (GO:0035688)
CD8-positive, alpha-beta T cell extravasation	alpha-beta T cell extravasation⋯(GO:0035697)
monocyte chemotaxis	monocyte chemotaxis (GO:0002548)
positive regulation of monocyte chemotaxis	pos. regulation of monocyte⋯(GO:0090026)
regulation of monocyte chemotaxis	regulation of monocyte chemotaxis (GO:0090025)
reactive oxygen species biosynthetic process	ROS biosynthetic process⋯(GO:1903409)
positive regulation of reactive oxygen species biosynthetic process	pos. regulation of ROS synthetic⋯(GO:1903428)
response to interleukin-1	response to IL-1 (GO:0070555)
interleukin-1 alpha production	IL-1 alpha production (GO:0032610)
negative regulation of interleukin-13 production	neg. regulation of IL-13⋯(GO:0032696)
negative regulation of interleukin-12 production	neg. regulation of IL-12⋯(GO:0032695)
positive regulation of interleukin-10 production	pos. regulation of IL-10⋯(GO:0032733)
platelet activation	platelet activation (GO:0030168)
regulation of platelet activation	regulation of platelet activation (GO:0010543)
platelet aggregation	platelet aggregation (GO:0070527)
regulation of blood coagulation	regulation of blood coagulation (GO:0030193)
complement activation	complement activation (GO:0006956)
blood coagulation, fibrin clot formation	blood coagulation⋯(GO:0072378)
DNA replication	DNA replication (GO:0006260)

DNA repair	DNA repair (GO:0006281)
regulation of DNA-dependent DNA replication	regulation of DNA replication···(GO:0090329)
nucleobase-containing compound catabolic process	nucleobase compound···(GO:0034655)
organic cyclic compound catabolic process	cyclic compound···(GO:1901361)
aromatic compound catabolic process	aromatic compound···(GO:0019439)
tRNA aminoacylation	tRNA aminoacylation (GO:0043039)
tRNA aminoacylation for protein translation	tRNA aminoacylation for protein···(GO:0006418)
TOR signaling	TOR signaling (GO:0031929)
sphingoid biosynthetic process	sphingoid biosynthetic (GO:0046520)
endosomal vesicle fusion	endosomal vesicle fusion (GO:0034058)
sphingolipid mediated signaling pathway	sphingolipid mediated signaling···(GO:0090520)
homeostasis of number of cells within a tissue	homeostasis of number of cells···(GO:0048873)
regulation of pro-B cell differentiation	regulation of pro-B cell···(GO:2000973)
pro-B cell differentiation	pro-B cell differentiation (GO:0002328)
lymphoid progenitor cell differentiation	lymphoid progenitor cell ···(GO:0002320)
B-1a B cell differentiation	B-1a B cell differentiation (GO:0002337)
B-1 B cell differentiation	B-1 B cell differentiation (GO:0001923)

Table S2. Mapping of RNA-seq reads to the reference genome (MesAur1.0) in six samples, related to Figure 5

Sample	Clean reads	Total mapped reads
Mock-1	22,598,170	20,153,048 (89.18%)
Mock-2	27,496,897	24,747,207 (90.00%)
SARS-CoV-2-1	24,312,770	21,533,820 (88.57%)
SARS-CoV-2-2	24,189,043	21,056,562 (87.05%)
SARS-CoV-2-Methotrexate-1	27,955,929	24,908,733 (89.10%)
SARS-CoV-2-Methotrexate-2	29,227,994	26,015,837 (89.01%)

Table S3. Gene-specific primers and probe sequences for hamster cytokine/chemokine mRNA profiling, related to Figure 5 List of probes and primers used in qRT-PCR assays.

Gene	Forward	Reverse
SARS-CoV-2 E gene	ACAGGTACGTTAATAGTTAATAGCGT	ATATTGCAGCAGTACGCACACA
	Probe FAM-ACACTAGCCATCCTTACTGCGCTTCGBBQ	
Hamster TNF-α	TTTGGAGTCATCGCTCTGTG	AACCGTTTGAATCCTTGACG
Hamster IFN-γ	CCATCAAGGCAGACCTGTTT	TTCTTGTTGGGACGATTTCC
Hamster IL-6	CTCCGCAAGAGACTTCCATC	ACCAAACCTCCGACTTGTTG
Hamster IL-10	CAAGGGACGCGTGGTAGTAT	GTCCTGAACTCAGGGATGGA
Hamster CCL22	CATCTGCTGCCAGGACTACA	TCTTCACCAGGCCAGCTTAT
Hamster PAI-1	CCGTGGAACCAGAACGAGAT	ACCAGAATGAGGCGTGTCAG
Hamster Actin	GTCGTACCACTGGCATTGTG	CCATCTCTTGCTCGAAGTCC

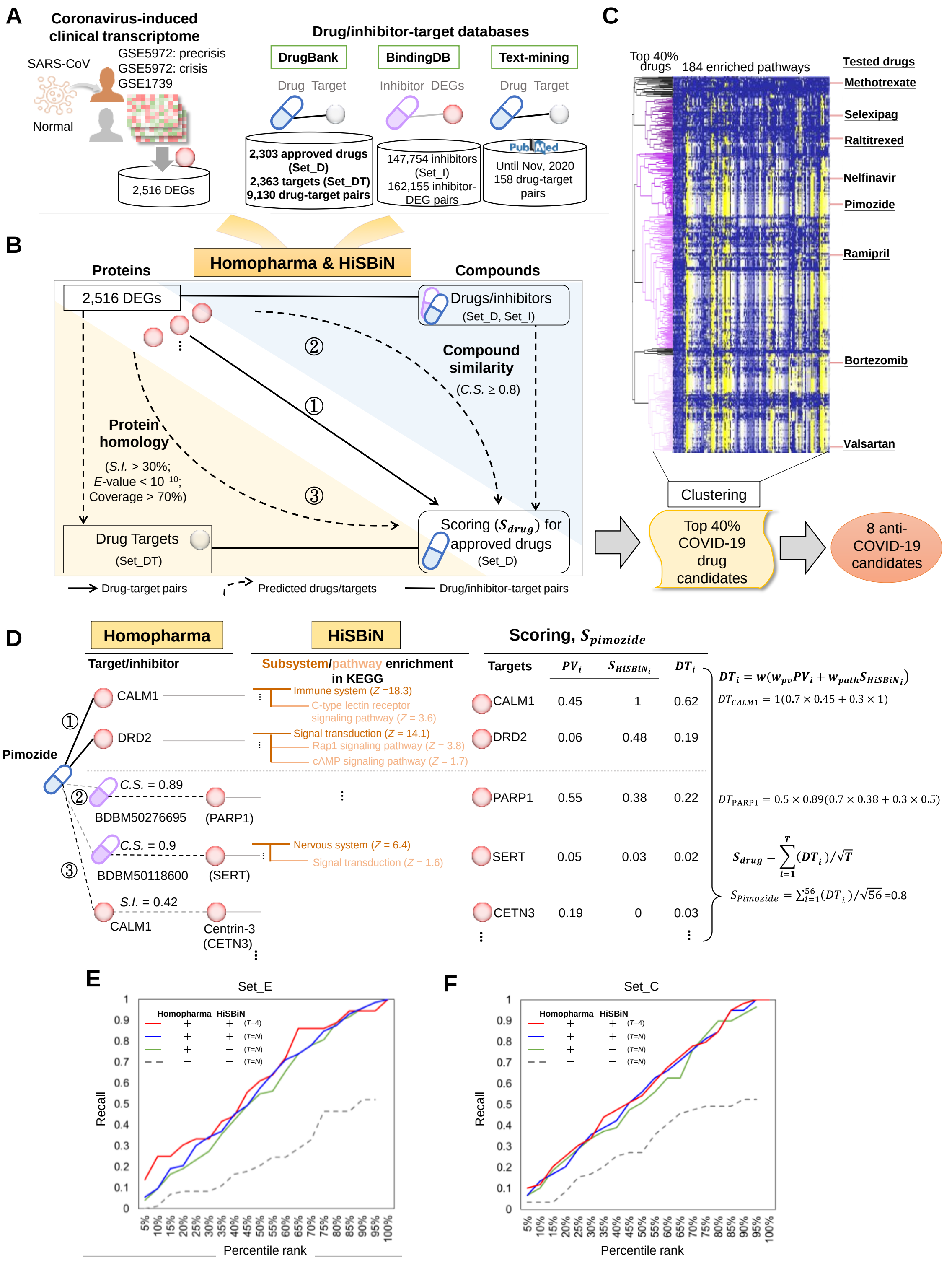

Figure S1. Combining Homopharma and HiSBiN for multi-target repurposing drugs

(A) Input datasets for the integrated scoring S_{drug} (Homopharma and HiSBiN), including disease-specific transcriptome (RNA-seq) derived DEGs and drug/inhibitor-target datasets collected from various databases. Clinical transcriptomes in three omics data sets (GSE5972: precrisis, GSE5972:

crisis and GSE1739) from human SARS-CoV-infected samples were analyzed to obtain a 2,516 DEGs set **(B)** Schematic description of the integrated platform steps using Homopharma, HiSBiN and drug scoring (S_{drug}) for discovering multi-target anti-COVID-19 drugs. The DEG-drug/inhibitor pairs (complexes) are first queried to identify multiple drug-target pairs through either of the following predictions: ① DEG-drug pairs recorded; ② DEG-inhibitor/compound (Set_D, Set_I) similar to approved drugs (compound similarity *C.S.>0.8*), ③ DEG homologous proteins (sequence identity *S.I.>30%*, BLASTP *E*-value ≤ 10^{-10}, aligned sequence coverage > 70%) that are drug targets (Set_DT). Thus, based on the predictions, all the drugs in Set_D were scored by S_{drug}, that comprises of multi-targeting and S_{HiSBiN}. **(C)** The top 40% of the scored drugs were clustered by hierarchical clustering based on their involvement in the enriched pathways. From each cluster, representative drugs were selected and 8 potential anti-COVID-19 drug candidates were further tested. **(D)** Predictions and drug scoring for the example multi-targeting drug, pimozide. The S_{drug} scoring is derived from the score of target protein *i* (DT_i) and the number of targets (*T*). DT_i is derived from the score of the average gene expressions of target *i* (PV_i) and its subsystems/pathways enrichment score S_{HiSBiN}. For pimozide, predicted targets, PARP1, SERT and CETN3 through *C.S.* and *S.I.* Then we applied HiSBiN to analyze the subsystem or pathway enrichment in KEGG for its multiple drug targets. For instance, we identified CALM1 associated in enriched subsystem, such as immune system (*Z* score, *Z* =18.3) and pathways, such as C-type lectin receptor signaling pathway (*Z* = 3.6). The scoring for pimozide $S_{Pimozide}$, determined using PV_i, S_{HiSBiN_i} and DT_i for its targets and their enriched subsystems and pathways, is 0.8. **(E and F)** Performance was evaluated using recall of the different percentile drug ranks with (+) or without (–) Homopharma and HiSBiN. **(E)** Reference Set_E has 77 drugs. **(F)** Reference Set_C has 58 drugs. *T* is the number of targets and *N* is total number of predicted targets for each drug.

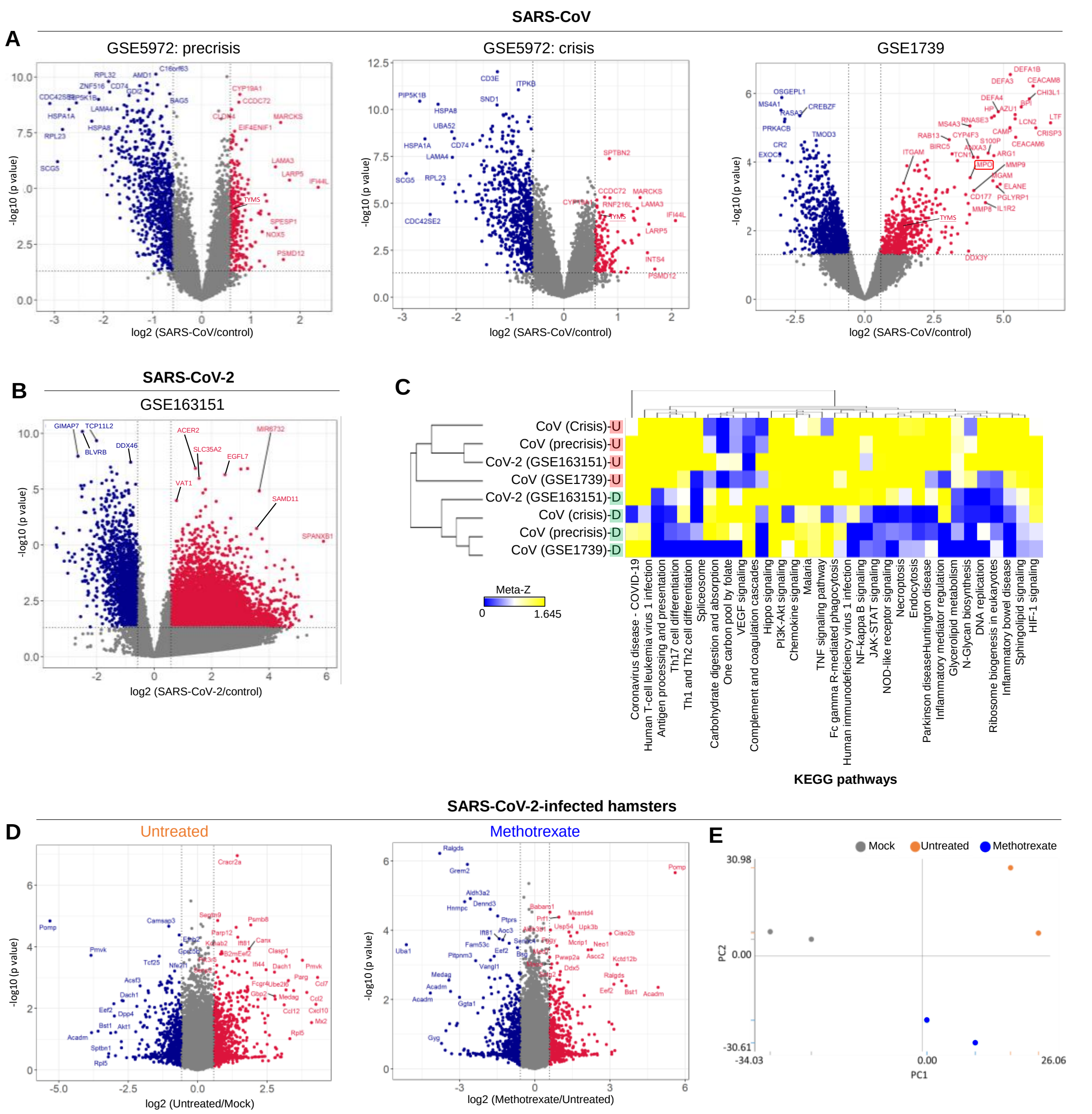

Figure S2. **Omics analysis of SARS-CoV and SARS-CoV-2 transcriptomic and animal model datasets**

Volcano plots for omics datasets, with the fold change (FC) and p value of genes between samples, and the identified differentially expressed genes (DEGs). **(A)** Analysis of public GEO SARS-CoV datasets (infected *vs.* control samples), all peripheral blood mononuclear cells (PBMCs) sampled from clinical SARS-CoV infected patients for GSE5972: precrisis, GSE5972: crisis and GSE1739 datasets. The cutoffs for FC > 2 and p value < 0.05 were applied to yield 1157, 830 and 1,440 DEGs for GSE5972: precrisis, GSE5972: crisis and GSE1739, respectively. Some specific DEGs such as COVID-19-related neutrophil MPO (outlined) in GSE1739 and methotrexate-related TYMS (underlined) are highlighted. **(B)** Analysis of SARS-CoV-2 public dataset GSE163151 (infected *vs.* control samples) from whole blood (WB) of clinical SARS-CoV-2 infected patients, identifies 3,673 DEGs. **(C)** Hierarchical clustering of SARS-CoV and SARS-CoV-2 public datasets and their DEG enrichment in pathways. **(D)** Analysis of in-house SARS-CoV-2-infected Syrian hamster lung RNA-seq data. In total, 710 upregulated and 582 downregulated DEGs were identified for untreated

case (untreated-infected *vs.* control samples), and 489 upregulated and 621 downregulated DEGs were identified by methotrexate-treated case (methotrexate-treated *vs.* untreated-infected samples). **(E)** Principal component analysis (PCA) to examine the data quality of the RNA-seq data from six in-house hamsters, including mock (gray), untreated (orange) and methotrexate-treated (blue) samples using WebMeV (Multiple Experiment Viewer). PC1 is the first principal component; PC2 is the second principal component. U (red) – upregulated DEG sets, D (green) – downregulated DEG sets, CoV: SARS-CoV; CoV-2: SARS-CoV-2; WB: whole blood. For (**A**), (**B**) and (**D**), red dots – upregulated DEGs, blue dots – downregulated DEGs.

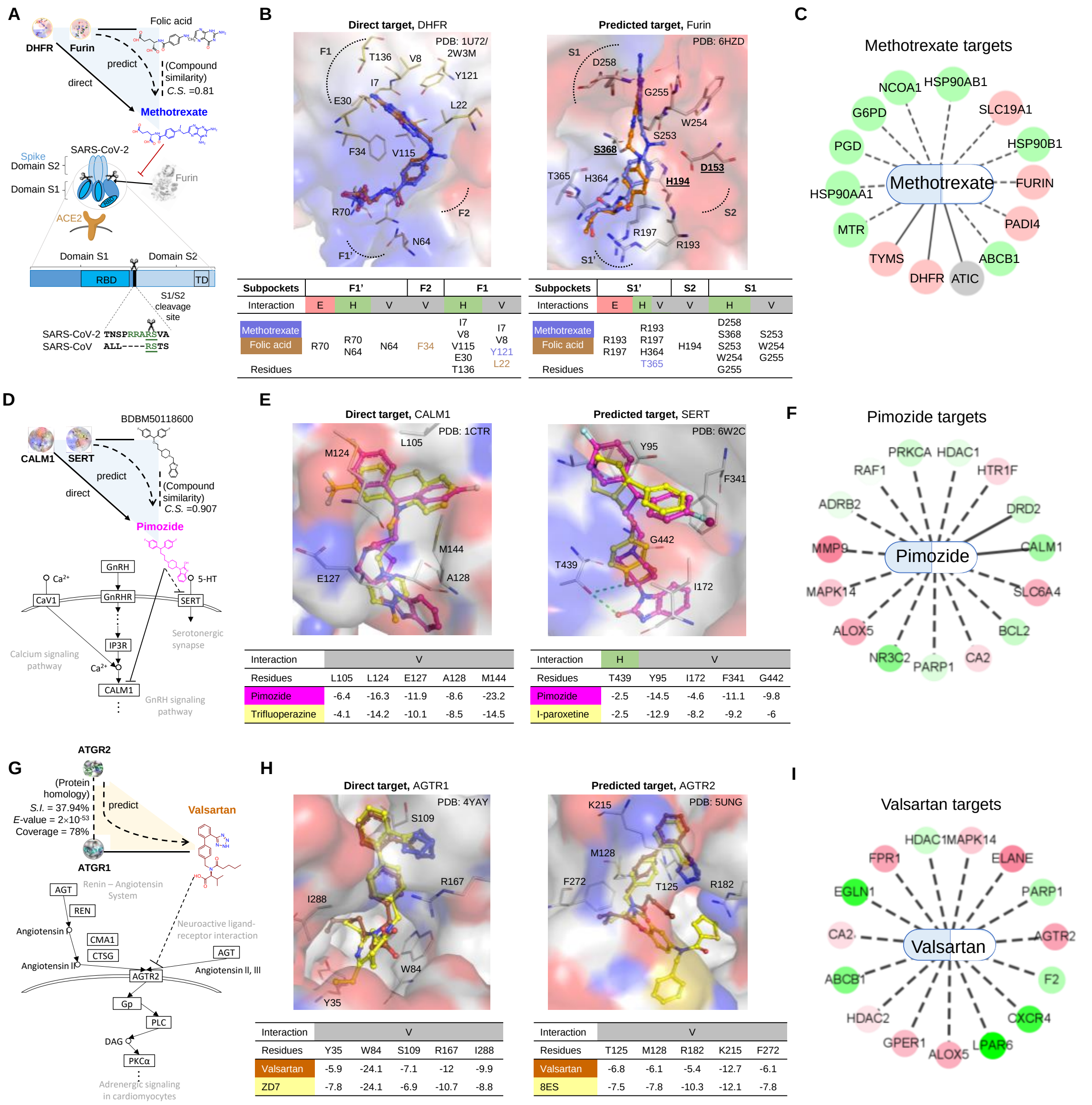

Figure S3. Case studies of methotrexate, pimozide, and valsartan

(A) Case of methotrexate. Methotrexate is similar to compound folic acid, *C.S.* = 0.81, and its direct target DHFR has similar binding environments with furin. Furin is a protease that cleaves the SARS-CoV-2 spike between S1/S2 domains (at the arg(R)-ser(S) peptide bond of substrate peptide

motif RRARS), leads to RBD release and binding to ACE2 facilitating viral entry, which is inhibited by methotrexate. The spike motifs in SARS-CoV-2 and reference SARS-CoV are shown. **(B)** Binding modes of methotrexate complexed in direct target DHFR (PDB: 1U72) aligned with the predicted target furin, along with folic acid complexed in direct target DHFR (PDB: 2W3M) and furin. Similar active site binding environments of DHFR and furin with corresponding F1, F1', F2 and S1, S1', S2 subpockets, respectively, and similar interactions by methotrexate/folic acid with the subpocket residues. The table summarizes the corresponding matched binding interactions of methotrexate and folic acid in DHFR and furin (some specific residue interactions colored purple blue – methotrexate and folic acid – orange). For example, methotrexate's positively charged pterin rings are fixed in the negatively charged F1/S1 subpockets, in DHFR engaged charged E30 and polar T136, in furin engaged charged D258 and polar S368. Also, its negatively charged carboxylic acid tail is at the positively charged F1'/S1' subpockets of DHFR/furin, binding to positive R193, R197 at S1' of furin matching with R70 at the F1' of DHFR. Protein surfaces are colored by their charge, blue: positive, red: negative. **(C)** Methotrexate targets by Homopharma including direct (solid line) and predict (dashed line) targets. **(D)** Case of pimozide, with a direct target, calmodulin-1 (CALM1), and a predicted target, sodium-dependent serotonin transporter (SERT) which was predicted, by high compound similarity (*C.S.* = 0.9) of a SERT inhibitor BDBM50118600 with pimozide. Also in KEGG, the two target proteins are involved in the GnRH signaling, calcium signaling and serotonergic synapse pathways. **(E)** Binding pose of pimozide (magenta) in CALM1 (PDB: 1CTR) with ligand trifluoperazine (yellow) and in SERT (PDB: 6W2C) with ligand I-paroxetine (yellow) are shown. In the interaction profile table, the similar interactions with key residues at target binding pockets for the docked pose and the ligand are displayed. For example, in CALM1, pimozide and the ligand showed matched V-interactions (with L105, L124 etc., gray); in SERT pimozide and the ligand showed matched H-bonding (with T439, green) and V-interactions (with Y95, I172 etc., gray). **(F)** Pimozide Top 15 targets by Homopharma including direct (solid line) and predict (dashed line) targets. **(G)** Case of valsartan, with a direct target, Type-1 angiotensin II receptor (AGTR1) and a predicted target, Type-2 angiotensin II receptor (AGTR2) with protein homology (*E*-value = 2E-53; sequence identity (*S.I.*) = 37.94% and coverage = 78%). Also in KEGG, the two target proteins are involved in the pathways of renin-angiotensin system, adrenergic signaling and neuroactive ligand-receptor interaction. **(H)** Binding poses of valsartan (brown) in AGTR1 (PDB: 4YAY) with ligand ZD7 (yellow) and in AGTR2 (PDB: 5UNG) with ligand 8ES (yellow) are shown. The interaction profile table describes the similar interactions with key residues at target binding pockets for the docked poses and the ligand. For example, in AGTR1, valsartan and the ligand ZD7 showed matched V-interactions (with Y35, W84 etc., gray); in AGTR2 valsartan and the ligand 8ES showed matched V-interactions (with T125, F272 etc., gray). **(I)** Valsartan Top 15 targets by Homopharma with predicted (dashed line) targets.

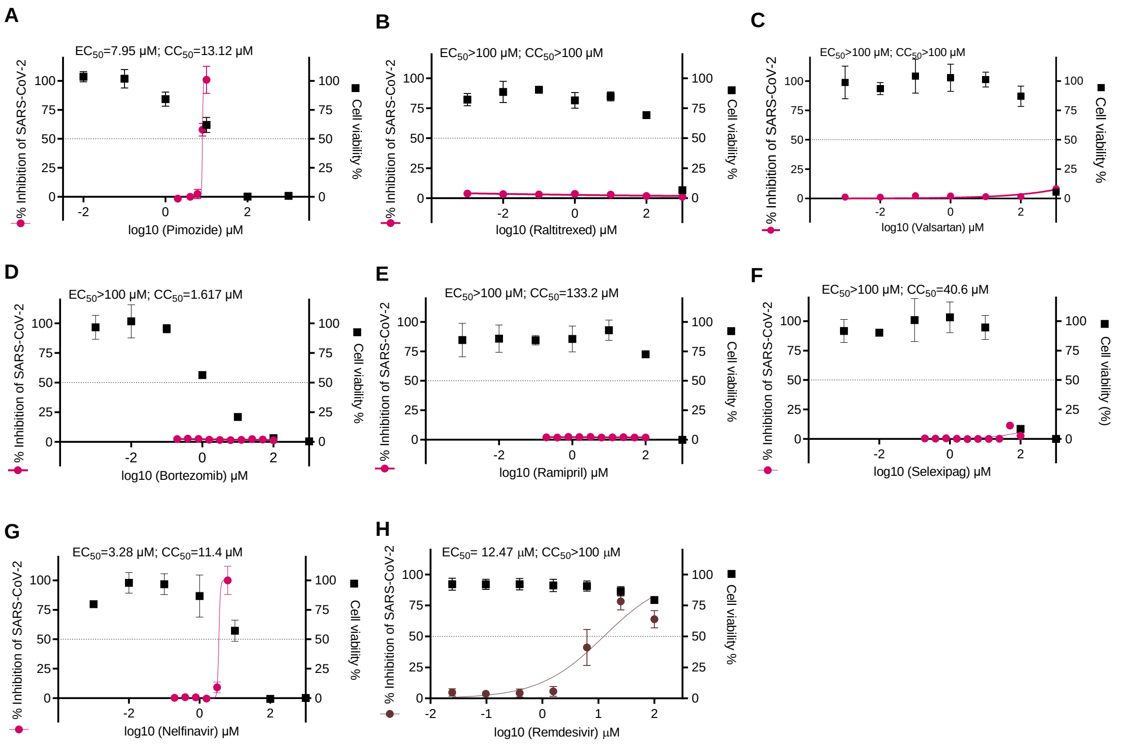

I

Drug name	Primary drug targets	FDA indication	Antiviral efficacy (EC_{50} in μM)	Cytotoxicity (CC_{50} in μM)
Methotrexate	Dihydrofolate reductase inhibitor	Rheumatoid arthritis	0.4	>100
Pimozide	Dopamine receptor blocker	Anti-psychotic	7.95	13.12
Raltitrexed	Thymidilyte synthase inhibitor	Anti-cancer	>100	>100
Valsartan	AT1 receptor blocker	Cardiovascular	>100	>100
Bortezomib	Proteasome inhibitor	Anti-cancer	>100	1.62
Ramipril	ACE inhibitors	Diabetic kidney disease	>100	133.2
Selexipag	Prostacyclin agonist	Pulmonary hypertension	>100	40.6
Nelfinavir	protease inhibitor	Human immunodeficiency virus (HIV)	3.28	11.4
Remdesivir	Virus RdRp inhibitor	Anti-COVID19	12.47	>100

Figure S4. Viral replication inhibition and cytotoxicity for the tested drug candidates

The inhibition of SARS-CoV-2 replication (red circles) and EC_{50} curves evaluated by CPE reduction assays, and the cell viabilities and CC_{50} curves by MTT assay for cell viability (black squares), for 8 drug candidates: **(A)** pimozide, **(B)** raltitrexed, **(C)** valsartan, **(D)** bortezomib, **(E)** ramipril, **(F)** selexipag, **(G)** nelfinavir and **(H)** the standard drug remdesivir (brown circles for inhibition). **(I)** A table listing the drug candidates and their *in vitro* EC_{50} and CC_{50} values.

For each DEG (i), we evaluate the disease gene score (S_{DisG_i}) of each DEG by its gene expression (S_{PV_i}), druggability ($S_{Druggability_i}$) and HisBiN (S_{HiSBiN_i})

A

DEG (i)	S_{PV_i}	$S_{Druggability_i}$	S_{HiSBiN_i}
PADI4	2.94	46	6.4
Normalize by all DEGs			
	0.15	0.28	0.427

$$S_{DisG_{PADI4}} = 0.5 \times S_{PV_{PADI4}} + 2 \times S_{Drugg abiliilty_{PADI4}} + 1 \times S_{HiSBiN_{PADI4}}$$
$$= 0.5 \times 0.15 + 2 \times 0.28 + 1 \times 0.427 = 1.057$$

B

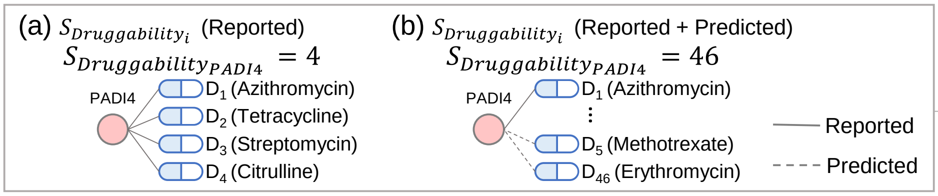

C

Score of a gene involvement in COVID-19 related subsystem/pathways (S_{HiSBiN_i})
(PADI4 as an example)

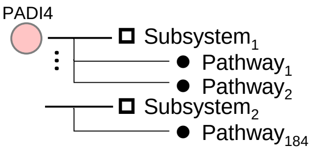

Z-score : transformed by enrichment *P* value

◻ Subsystem	Z score (Z_k)	Gene*
◻ Translation	0	✘
◻ Signal transduction	0	✘
◻ Transport and catabolism	0	✘
◻ Immune system	2.29	✔
◻ Endocrine system	0	✘
◻ Circulatory system	0	✘
⋮	⋮	⋮
◻ Infectious disease: viral	0	✘

* ✔: Gene-involved in ✘: Non gene-involved in

● Pathway	Z-score (Z_j)	Gene*
• mRNA surveillance pathway	4.2	✘
• JAK-STAT signaling pathway	2.3	✘
• TNF signaling pathway	3.9	✘
• Endocytosis	3.9	✘
• Neutrophil extracellular trap formation	10.5	✔
• Natural killer cell mediated cytotoxicity	4.5	✘
• NOD-like receptor signaling pathway	4.51	✘
• Platelet activation	2.6	✘
⋮	⋮	⋮
• Antigen processing and presentation	6.68	✘

* ✔: Gene-involved in ✘: Non gene-involved in

$$Z_{Sub_{PADI4}} = \frac{\sum_{k=1}^{m} Z_k}{\sqrt{m}} = \frac{2.29}{\sqrt{1}} = 2.29$$

m : numbers of a gene-involved subsystems (here the maximum is 38)

$$Z_{Path_{PADI4}} = \frac{\sum_{j=1}^{n} Z_j}{\sqrt{n}} = \frac{10.5}{\sqrt{1}} = 10.5$$

n : numbers of a gene-involved in 184 COVID-19 KEGG pathways (here the maximum is 184)

$$S_{HiSBiN_{PADI4}} = \frac{Z_{Subs} + Z_{Path}}{2} = \frac{2.29+10.5}{2} = 6.4$$

Figure S5 Integration of Homopharma and HiSBiN for disease gene score (S_{DisG}). For gene PADI4, the calculation of **(A)**, $S_{DisG_{PADI4}}$, **(B)**, of $S_{Druggability_{PADI4}}$, **(C)**, $S_{HiSBiN_{PADI4}}$.

A

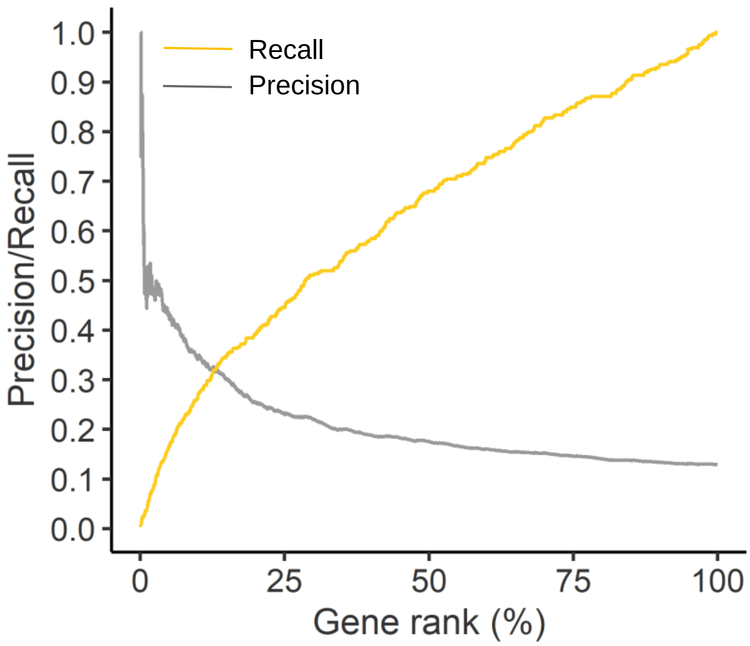

B

Gene name	S_{DisG}	Rank	Validation
JAK1	3.12	1	Reported in DisGeNET COVID-19 associated
GSK3B	3.08	2	Reported in DisGeNET COVID-19 associated
HDAC2	3.01	3	Reported in DisGeNET COVID-19 associated
HDAC1	3.01	4	PMID: 33398248
MAPK14	3.01	5	Reported in DisGeNET COVID-19 associated
IGF1R	3.00	6	Reported in DisGeNET COVID-19 associated
PIK3CB	2.91	7	Reported in DisGeNET COVID-19 associated
RAF1	2.79	8	Reported in DisGeNET COVID-19 associated
LCK	2.76	9	PMID: 34414199
PARP1	2.72	10	Reported in DisGeNET COVID-19 associated

Figure S6 Performance analysis for the disease gene score ($\boldsymbol{S_{DisG}}$).

(A) Relationships between recall (yellow) and precision (gray) for S_{DisG} values of 2,516 DEGs. Precision and recall are defined as TP/(TP+FP) and TP/(TP+FN) respectively, where TP, FP, and FN are the numbers of true-positive, false-positive, and false-negative cases. The highest F1 score of 0.38 was observed when S_{DisG} was set to 0.737. **(B)** Top 10 genes scored by $\boldsymbol{S_{DisG}}$ with validated references.

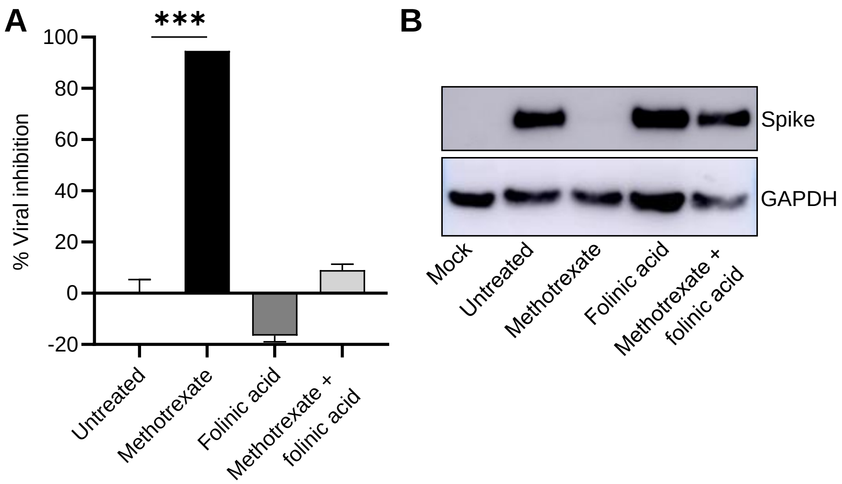

Figure S7. **Folinic acid rescue of viral replication inhibited by methotrexate**

(A) Folinic acid rescue of SARS-CoV-2 replication inhibition by methotrexate was performed infected Vero E6 cells, to examine that the anti-viral mechanism of action of methotrexate is by inhibition of DHFR leading to folate depletion. The experiments were repeated three times, and a two-sided p value was calculated with Student's *t*-test, ***p < 0.001, mean ± SD are shown. **(B)** From the infected and treated Vero E6 cells in **A**, cell lysates were collected and western blot analysis was performed. Viral protein synthesis reduced in methotrexate treatment (due to inhibition of DHFR, folate synthesis and viral replication) and was reversed and rescued by the addition of folinic acid.
